# Contactless Conductivity Detection for Capillary Electrophoresis—Developments From 2020 to 2024

**DOI:** 10.1002/elps.202400217

**Published:** 2024-11-28

**Authors:** Peter C. Hauser, Pavel Kubáň

**Affiliations:** ^1^ Department of Chemistry University of Basel Basel Switzerland; ^2^ Institute of Analytical Chemistry of the Czech Academy of Sciences Brno Czech Republic

**Keywords:** capacitively coupled contactless conductivity detection, capillary electrophoresis, microchip electrophoresis, review

## Abstract

The review covering the development of capillary electrophoresis with capacitively coupled contactless conductivity detection from 2020 to 2024 is the latest in a series going back to 2004. The article considers applications employing conventional capillaries and planar lab‐on‐chip devices as well as fundamental and technical developments of the detector and complete electrophoresis instrumentation.

**Abbreviations**
3‐HB: 3‐hydroxybutyrate
CF: cystic fibrosis
DBS: dried blood spot
DUS: dried urine spot
EBC: exhaled breath condensate
EME: electromembrane extraction
FASS: field amplified sample stacking
FDM: fused deposition modelling
FIA: flow injection analysis
FS: fused silica
GHB: γ‐hydroxybutyric acid
IC: ion chromatography
MD: microdialysis
NASA: National Aeronautics and Space Administration
PEEK: polyether ether ketone
PLA: polylactic acid
SI: sequential injection
UA‐DLLME: ultrasound‐assisted dispersive liquid–liquid microextraction
UV: ultra‐violet

## 1 Introduction

This review is the latest in a series of now 10 comprehensive summaries on the topic produced by the authors, which together cover the developments of capacitively coupled contactless conductivity detection (C^4^D) since its introduction to CZE in 1998. A number of reviews, generally focussing on C^4^D fundamentals, applications and special topics, have also been written by several other authors. Notably, Elbashir and Aboul‐Enein have written a series of reviews on pharmaceutical and biomedical applications of CE‐C^4^D. The reviews on C^4^D known to us are listed in Table 1, which offers the first comprehensive summary of all review articles published from 2001 to mid‐2024.

**TABLE 1:** Reviews on C^4^D.

Year	Corresponding author	Title	Comments	refs.
2001	Zemann	Conductivity detection in capillary electrophoresis	First and brief summary	[1]
2002	Hauser	Conductimetric and potentiometric detection in conventional and microchip capillary electrophoresis	Also includes contacted detection in CE	[2]
2003	Zemann	Capacitively coupled contactless conductivity detection in capillary electrophoresis	Summarizes all early developments	[3]
2004	Hauser	Contactless conductivity detection in capillary electrophoresis: A review	First in series by the current authors	[4]
2004	Haddad	Conductivity detection for conventional and miniaturized capillary electrophoresis systems	Also includes contacted detection in CE	[5]
2005	Guan	Capacitively coupled contactless conductivity detection in capillary electrophoresis	In Chinese language	[6]
2006	Kašička	Recent applications of conductivity detection in capillary and chip electrophoresis	Also includes contacted detection in CE	[7]
2007	Pumera	Contactless conductivity detection for microfluidics: Designs and applications	Focussed on lab‐on‐chip devices	[8]
2008	Hauser	A review of the recent achievements in capacitively coupled contactless conductivity detection	Second in series by the current authors	[9]
2008	Matysik	Advances in amperometric and conductometric detection in capillary and chip‐based electrophoresis	Also includes amperometric detection	[10]
2009	Hauser	Ten years of axial capacitively coupled contactless conductivity detection for CZE—a review	Third in series by the current authors	[11]
2010	Aboul‐Enein	Applications of capillary electrophoresis with capacitively coupled contactless conductivity detection (CE‐C^4^D) in pharmaceutical and biological analysis	First in series by Aboul‐Enein	[12]
2010	Paull	Non‐invasive characterization of stationary phases in capillary flow systems using scanning capacitively coupled contactless conductivity detection (sC^4^D)	Material characterization by C^4^D	[13]
2011	Hauser	Capacitively coupled contactless conductivity detection for microseparation techniques—recent developments	Fourth in series by the current authors	[14]
2012	Aboul‐Enein	Recent advances in applications of capillary electrophoresis with capacitively coupled contactless conductivity detection (CE‐C^4^D): an update	Second in series by Aboul‐Enein	[15]
2012	Fracassi da Silva	Capacitively coupled contactless conductivity detection on microfluidic systems—ten years of development	Focussed on lab‐on‐chip devices	[16]
2012	Matysik	Electrochemical methods in conjunction with capillary and microchip electrophoresis	Also includes amperometric detection	[17]
2013	Hauser	Contactless conductivity detection for analytical techniques: Developments from 2010 to 2012	Fifth in series by the current authors	[18]
2014	Aboul‐Enein	Recent applications and developments of capacitively coupled contactless conductivity detection (CE‐C^4^D) in capillary electrophoresis	Third in series by Aboul‐Enein, pharmaceutical, biomedical and food analysis	[19]
2015	Hauser	Contactless conductivity detection for analytical techniques—Developments from 2012 to 2014	Sixth in series by the current authors	[20]
2016	Pham	Inexpensive and versatile measurement tools using purpose‐made capillary electrophoresis devices coupled with contactless conductivity detection: A view from the case study in Vietnam	On CE‐C^4^D instruments	[21]
2017	Aboul‐Enein	Application of capillary electrophoresis with capacitively coupled contactless conductivity detection (CE‐C^4^D): An update	Fourth in series by Aboul‐Enein, pharmaceutical, biomedical and food analysis	[22]
2017	Hauser	Contactless conductivity detection for analytical techniques—Developments from 2014 to 2016	Seventh in series by the current authors	[23]
2018	Hauser	20th anniversary of axial capacitively coupled contactless conductivity detection in capillary electrophoresis	Broad overview with historical perspective in TrAC	[24]
2018	Van Schepdael	Recent advances in the capillary electrophoresis analysis of antibiotics with capacitively coupled contactless conductivity detection	Focus on antibiotics analysis	[25]
2019	Hauser	Contactless conductivity detection for analytical techniques: Developments from 2016 to 2018	Eight in series by the current authors	[26]
2020	Kubáň	Capacitively coupled contactless conductivity detection for analytical techniques—Developments from 2018 to 2020	Ninth in series by the current authors	[27]
2021	Tůma	Determination of amino acids by capillary and microchip electrophoresis with contactless conductivity detection—Theory, instrumentation and applications	Focus on amino acid analysis	[28]
2022	Tůma	Monitoring of biologically active substances in clinical samples by capillary and microchip electrophoresis with contactless conductivity detection: A review	Focus on clinical sample analysis	[29]
2022	Tůma	Capillary and microchip electrophoresis with contactless conductivity detection for analysis of foodstuffs and beverages	Focus on food analysis	[30]
2022	Aboul‐Enein	Application of capillary electrophoresis with capacitively coupled contactless conductivity detection (CE‐C^4^D): 2017–2020	Fifth in series by Aboul‐Enein, pharmaceutical, biomedical and food analysis	[31]
2024	Aboul‐Enein	Application of capillary electrophoresis with capacitively contactless conductivity detection for biomedical analysis	Sixth in series by Aboul‐Enein	[32]
2024	Dasgupta	Liquid phase detection in the miniature scale. Microfluidic and capillary scale measurement and separation systems: A tutorial review	Contains a discussion on fundamentals of C^4^D	[33]
2024	Maliushevska	Determination of amino acids and peptides without their pre‐column derivatization by capillary electrophoresis with ultraviolet and contactless conductivity detection: An overview	Also includes UV–Vis detection	[34]
2024	Kirsanov	Evolution of contactless conductometry methods	Various contactless concepts, including C^4^D	[35]

Readers interested in learning about the fundamentals may want to consult the basic studies published by the current authors in 2004 [36, 37] and by do Lago and co‐workers in 2005 [38, 39]. Tůma and co‐workers [40, 41, 42] as well as Dasgupta and co‐workers [43, 44, 45] have also published several studies on fundamentals, in particular on the effect of small inner diameters of capillaries (down to 10 µm or less), on the behaviour of C^4^Ds. Warren and Dasgupta in 2024 also published a tutorial review on microfluidic scale analysis, which contains an insightful discussion of conductivity detection [33].

Most of the about 170 publications on C^4^D from the recent 5 years concern applications rather than fundamental developments. CE‐C^4^D has mainly been used in the analyses of clinical, pharmaceutical, food and environmental samples, and this has followed the trends from previous years. The majority of applications have employed capillary electrophore (CE) in conventional capillaries, whereas the reported uses on lab‐on‐chip devices have mostly been relatively simple analytical tasks, a recent trend being the use of commercial glass microchips with embedded C^4^D electrodes. A fair number of studies on the use of C^4^Ds in analytical techniques other than CE appeared, demonstrating its usefulness in high‐performance liquid chromatography (HPLC), ion chromatography (IC), flow injection analysis (FIA), paper‐based microfluidics and other flow‐through methods. A number of reports on fundamental or technical developments of the detector and complete electrophoresis instrumentation or lab‐on‐chip devices incorporating C^4^D have still appeared. Below, these fundamental aspects are covered first, followed by the applications. Note that articles might be mentioned repeatedly in different sections if they relate to aspects discussed separately. Generally, the detailed discussion covers only C^4^D in CE, but any omissions of publications from within this topic are inadvertent, and we apologize for any oversights.

## 2 Fundamental Characterization and Instrumental Developments

### 2.1 C^4^D for Conventional Capillaries

Huhn and co‐workers [46] carried out an expanded study on the previously reported approach [47, 48, 49] for signal acquisition using an integrated circuit (AD7745 from Analogue Devices), which was designed for measuring small capacitances. This integrated circuit applies voltage pulses to a device under test and measures the resulting flow of electrical charge. Although the integrated circuit is sold as a capacitance‐to‐digital convertor, it can also be applied to a C^4^D as in this case the conductivity of the solution determines the current. Huhn and co‐workers [46] improved the approach by adding an auxiliary amplifier to boost the excitation voltage and also implemented wireless data transmission. The performance was reported to be similar to a commercially available detector (www.eDAQ.com) and the open‐source C^4^D by Mendonca Francisco and do Lago [50]. The design by Huhn and co‐workers is also available as open‐source hardware (https://github.com/AGHuhn/CDC_C4D).

Zeng and Pu with co‐workers [51] reported a detailed study on a state‐of‐the‐art differential C^4^D employing two electrode pairs, which allows for compensation of temperature drifts and noise [52, 53, 54]. Very low limits of detection (LODs) were reported for water samples, but note that the sensitivity in CE‐C^4^D is also dependent on the background conductivity of the samples and the injection parameters, and comparable LODs will not be achievable for all applications. The authors subsequently reported a different version of their detector where the benchtop units employed in their earlier set‐up (function generator, lock‐in amplifier) were replaced by a compact dedicated electronic circuitry, employing a DDS (direct digital synthesis) integrated circuit for sine wave generation and an RMS‐to‐DC (root mean square to direct current) convertor instead of the lock‐in amplifier [55]. Itterheimová et al. [56] reported a low‐cost data acquisition system for a C^4^D, which was based on an Arduino Nano microcontroller platform with an attached high‐resolution analogue‐to‐digital converter integrated circuit. The digitized reading was sent by the Arduino microcontroller via USB to a PC where it was imported directly into an Excel spreadsheet. Ferreira Santos et al. [57], in view of its deployment in space exploration, demonstrated that some of the semiconductors required for the circuitry of a C^4^D could be replaced with radiation‐hardened electronic components.

Zhang and co‐workers have reported several detectors that combine C^4^D with optical measurements at a common point. The first of these featured visible light‐emitting diodes (LEDs) for absorbance measurements of metal ion complexes with 4‐(2‐pyridylazo)resorcinol (PAR) [58]. A second detector featured ultra‐violet (UV)‐LEDs at 255 or 235 nm and was demonstrated for the indirect absorption detection of a range of compounds [59]. A combination cell for laser‐induced fluorescence (LIF) and C^4^D was demonstrated for inorganic cations (C^4^D) and fluorescein isothiocyanate‐derivatized amino acids (LIF) [60]. Finally, the authors presented a triple detector featuring LIF and UV absorption besides C^4^D [61].

**FIGURE 1:**
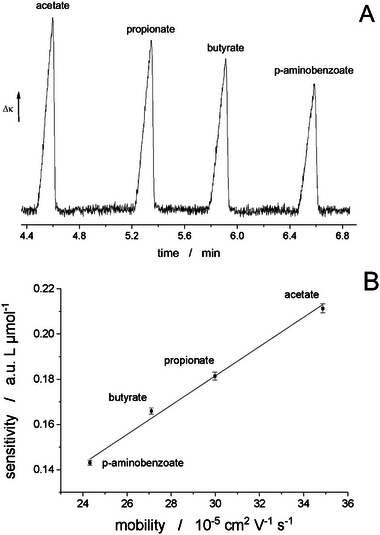
(A) Electropherogram for four anions. (B) Plot of the sensitivity for these ions against their mobility. *Source*: Reproduced from *Analytica Chimica Acta* [62] with permission from Elsevier.

Santana and do Lago [62] studied calibration in CE‐C^4^D. Because the sensitivity for every analyte depends on its molar conductivity, each analyte normally requires a specific calibration. The authors showed that calibration curves can be extended to a range of species by employing interpolation. This implies that quantification is also possible for analytes, for which a standard is not available. In Figure 1, the interpolation procedure is illustrated by showing the dependence of the sensitivity for several carboxylate anions on the mobility of these ions. The approach should be highly useful for practitioners of CE‐C^4^D. Tong et al. [63] studied different numerical approaches for the processing of CE‐C^4^D electropherograms, post‐acquisition, in order to reduce noise. They proposed a method termed ‘improved wavelet threshold function’, but its applicability to lowering LODs has not been demonstrated.

### 2.2 Purpose‐Made CE‐C^4^D Instruments

**FIGURE 2:**
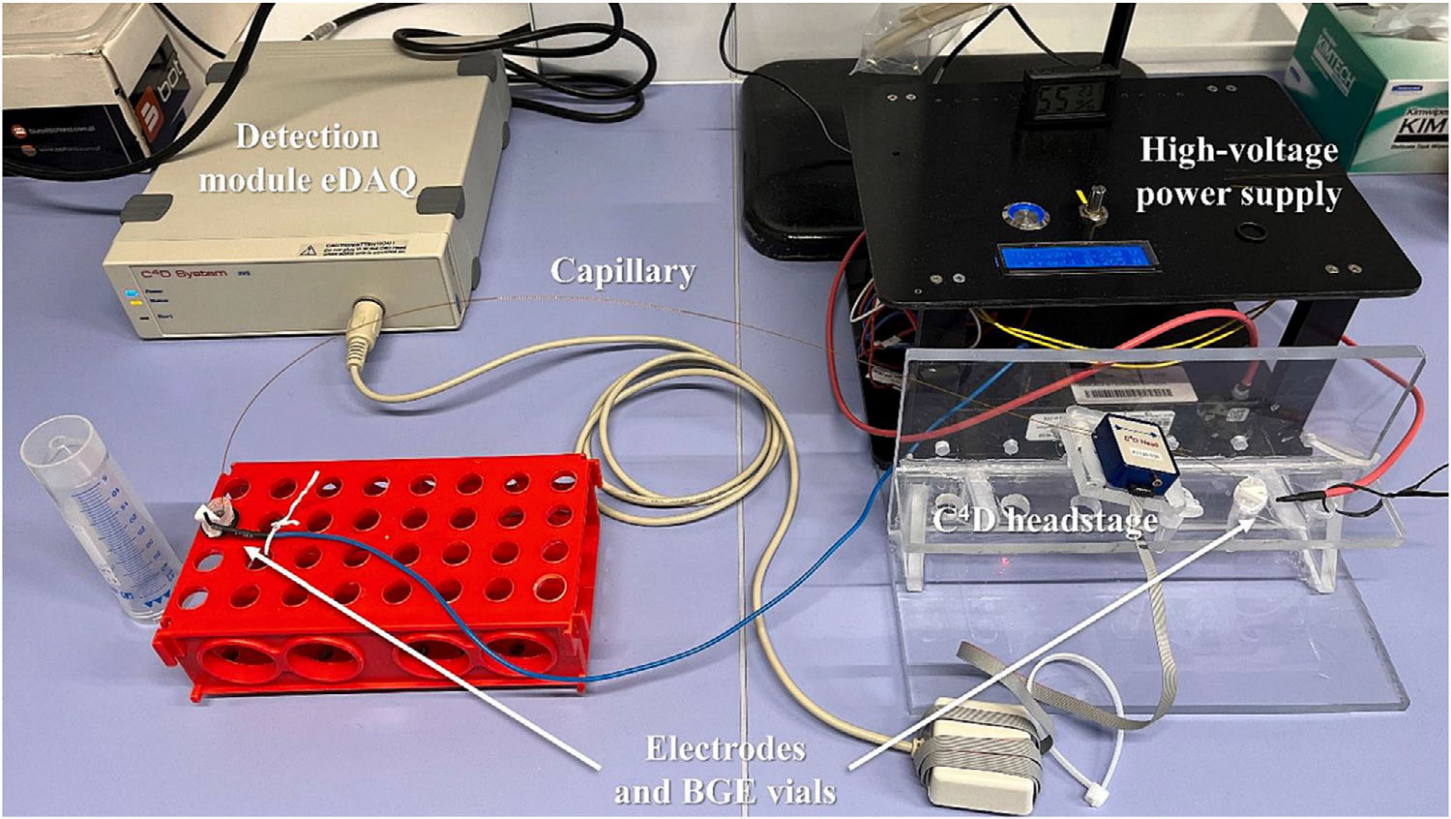
Photograph of a purpose‐made manually operated instrument based on a commercial C^4^D. BGE, background electrolyte. *Source*: Reproduced from *Microchemical Journal* [68] with permission from Elsevier.

Commercial CE instruments offer high repeatability of analytical procedures (injected volumes, migration times), excellent baseline stability, acceptable sensitivity and good instrument‐to‐instrument transferability. However, the cost of a commercial benchtop CE instrument is usually high (e.g., nearly €100 000 for an Agilent 7100 CE) and is not affordable for every laboratory. Instead of commercial instruments, significantly less costly lab‐made instruments may be used; however, building an automated CE system requires electronic skills and/or the help of specialized workshops, which will not always be available. Furter et al. have, thus, suggested a modular CE system assembled from commercially available components, which are (i) easily available, (ii) affordable and (iii) their assembly does not require the involvement of a mechanical workshop [64]. The only special building task required was the fabrication of an electronic circuitry for controlling the instrument. Liquid handling and sample injections were achieved with the help of compressed nitrogen from a standard gas cylinder. System control was based on an Arduino and a user interface on a PC written in Java. Various features of a lab‐made autosampler for injections into portable CE instruments were comprehensively investigated by Kaljurand and co‐workers [65]. With a set of peristaltic and vacuum pumps, pinch and check valves, and standard tubing, an inexpensive sampler was fabricated. Itterheimová and Kubáň [66] reported a purpose‐made low‐cost CE‐C^4^D instrument with a rotary autosampler of their design as part of the system, addressing an important practical aspect not often considered in the construction of purpose‐made instruments. Zhang et al. [67] reported a dedicated CE‐C^4^D instrument for the on‐site determination of the macronutrients (nitrate, ammonium, phosphate and potassium) in soil extracts. Their C^4^D was assembled from individual modules for the different functions of the circuitry. Rusin et al. [68] reported a very simple manually operated instrument employing a commercially available C^4^D. This is illustrated in Figure 2. Wang et al. [69] described a compact portable CE‐C^4^D instrument, for which a smartphone connected via Bluetooth was employed as a control and data acquisition device. A portable CE‐C^4^D instrument with a pressure‐driven injection module for on‐site ionic analysis was constructed by Zhang and co‐workers [70]. Sample injection and capillary flushing were again accomplished with compressed nitrogen from a gas cylinder, and samples were mixed with an internal standard and introduced into the capillary through a 6‐port injection valve. The system was tested for continuous autonomous CE‐C^4^D analyses of inorganic anions and cations in water samples for a period of 35 h.

**FIGURE 3:**
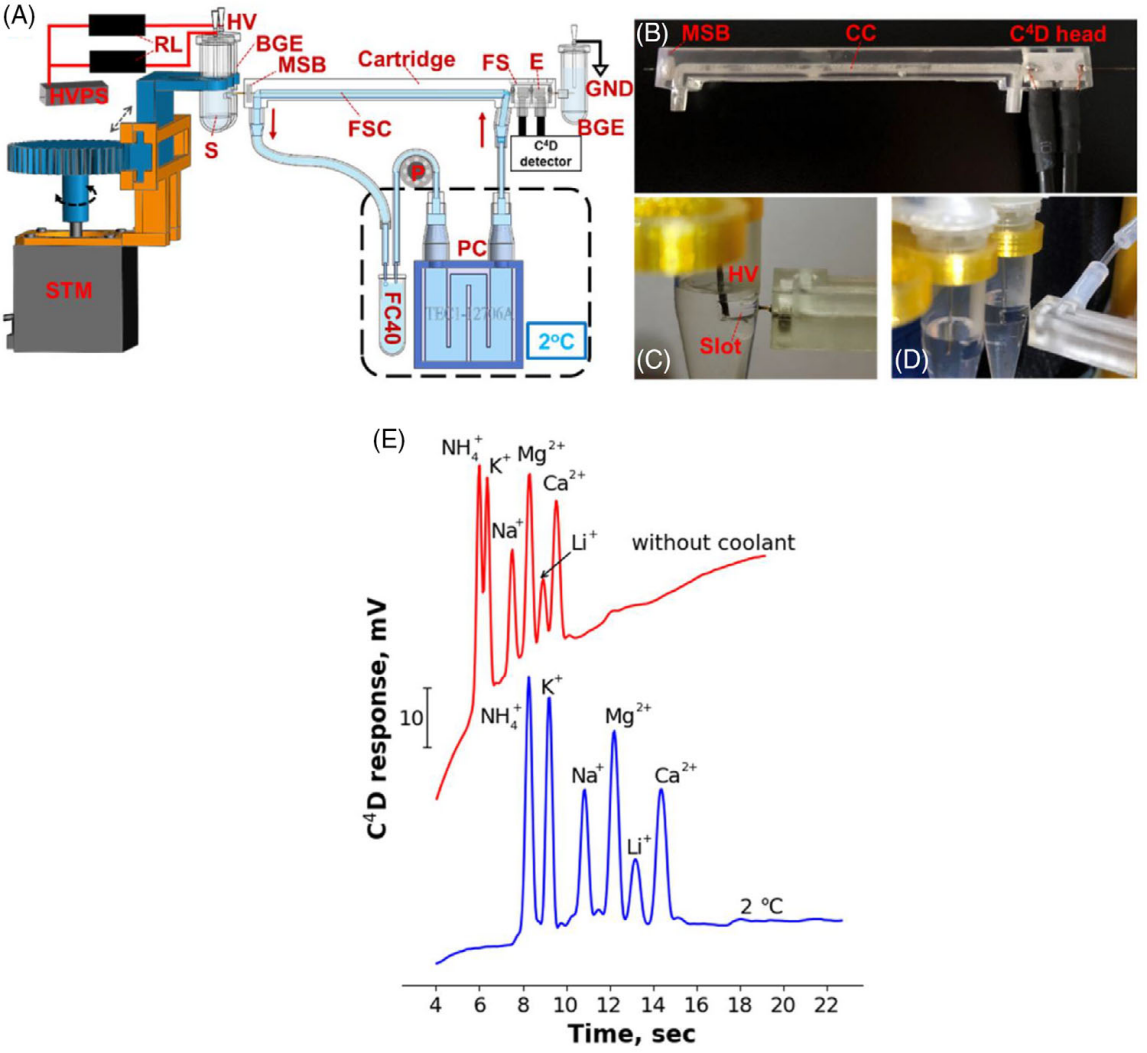
Schematics (A), photographs (B‐D) and the effect of external cooling using a 3D printed cartridge, visualized by fast CE‐C^4^D of inorganic cations (E). BGE, background electrolyte; CC, coolant channel; E, electrode; FC40, coolant; FS, Faraday shield; FSC, fused silica capillary; GND, ground; HV, high voltage; HVPS, high voltage power supply unit; MSB, metal sealing block; P, peristaltic pump; PC, Peltier cooler; RL, high voltage reed relay; S, sample; STM, stepper motor. *Source*: Reproduced from *Analytica Chimica Acta* [71] with permission from Elsevier.

Zhang and co‐workers also designed a 3D‐printed cartridge for high‐speed CE‐C^4^D analyses with the temperature control of the separation capillary [71]. In high‐speed CE, the separations are performed typically at voltages ≥1000 V/cm in short capillaries and result in the generation of excessive Joule heat. This, in turn, induces unstable baseline signals and, thus, worsens CE stability, repeatability and sensitivity. The authors have demonstrated that the cartridge and the external cooling to 2°C significantly improved the analytical parameters, and the proposed concept was used for rapid separations (in ≤22 s) of inorganic cations and anions in drinking water samples and black tea extracts. The cartridge also included a C^4^D cell created by filling channels with molten Wood's metal. The photograph of the 3D‐printed cartridge, the main CE‐C^4^D components and the effect of the coolant on the separation of inorganic cations are depicted in Figure 3.

A commercial portable CE‐C^4^D system, which has been developed by the Australian company Grey Scan (Braybrook, Victoria, Australia: https://greyscandetection.com) in collaboration with Breadmore's group at the University of Tasmania, has become available. The instrument has been designed for the detection of inorganics used in improvised explosive devices and is discussed in Section 3.1.3. CE‐line (Emmeloord, The Netherlands: https://ce‐line.com), a Dutch company, has started selling a process analyser for the monitoring of nutrient solutions in greenhouses. The instrument was developed jointly with Hauser's group at the University of Basel and is capable of running autonomously for extended periods of time.

Drones are suitable for carrying objects in the air. Drones that can hover (being in a stagnant position in the air) are most attractive for analytical applications because they enable air sampling at a single spot. However, hovering drones are only suitable for carrying light‐weight (not more than 5 kg) objects, and most analytical instruments are by far too heavy and bulky to be carried by drones. Nevertheless, dimensions of portable CE instrumentation have been reduced in recent years, and Drevinskas et al. down‐scaled CE to a size/weight compatible with hovering drones [72]. A complete CE‐C^4^D instrument, including a device for air sampling, could be accommodated on a commercial drone.

**FIGURE 4:**
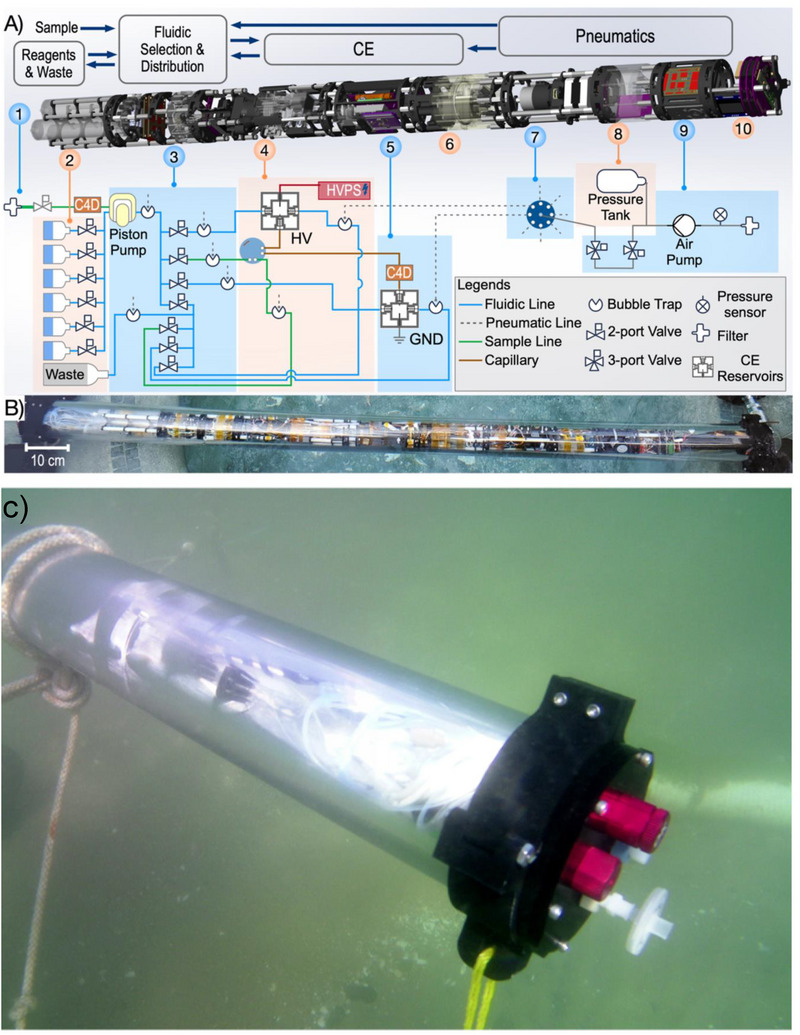
Schematics (A), photograph (B) and the underwater operation of a submersible CE‐C^4^D (C). HV, high voltage; HVPS, high voltage power supply unit; GND, grounding. *Source*: Reprinted from *Analytical Chemistry* ref. [74] with permission from American Chemical Society.

The group of Peter Willis and other researchers at the Jet Propulsion Laboratory presented several studies on instrumentation for potential employment of CE‐C^4^D in space missions. A fully automated proof‐of‐concept system with multiple detectors (LIF and MS besides C^4^D) for detecting small molecules of interest in astrobiology, that is, in the search for extra‐terrestrial life, was developed [73]. By the combination of CE separations with all three detection modes, a full range of analytes important for the search for chemical signatures of life can be monitored, including inorganic ions and amino acids (C^4^D), trace amino acids (LIF) and unknown species (MS). In a different project, a prototype of a submersible instrument suitable for fully unmanned sample collection, injection and separation underwater was designed with the view of bringing such an instrument to the ocean worlds of some of the moons of outer planets of our solar system [74]. Figure 4 shows the crucial components of the submersible CE‐C^4^D system and an underwater photograph of the instrument in a test operation in the Pacific Ocean. As the high voltage employed in CE can potentially cause problems in a spacecraft, a study was also carried out to determine by how much the applied separation voltage can be lowered without sacrificing the analytical performance (15 kV was deemed acceptable) [75]. Berg et al. developed a temperature‐control unit for separation capillaries as proof‐of‐concept for a spaceflight CE‐C^4^D [76]. The capillary was wound around two ceramic heat‐conducting elements in a metallic case, which was filled with thermally conductive epoxy resin. The temperature near the capillary was found to be highly stable even when fluctuating the external temperature over a range of 40°C.

### 2.3 Microchip Electrophoresis

Electrophoresis chips with integrated C^4^D electrodes for contactless detection are now readily available commercially from Micronit (Enschede, The Netherlands: https://micronit.com/) and were designed to fit into a matching holder with detector electronics from eDAQ (Denistone East, NSW, Australia: www.eDAQ.com). Applications of this platform are discussed in Section 3.2. However, these glass chips are expensive (ca. €400 each), and many recent reports concern alternatives to the lithographic processing of glass. 3D printing has been suggested as a suitable alternative for the fabrication of ME‐C^4^D devices. Additive manufacturing, unlike subtractive microfabrication techniques, does not require master masks or moulds, and 3D printing can directly produce microfluidic designs from CAD files. Moreover, modern 3D printers are affordable and can combine the printing of various materials [77]. This might be especially attractive for ME‐C^4^D, which may benefit from the simultaneous use of inert (plastic and translucent resins) and conductive (metals or composites) materials for 3D printing the microfluidic channels and the detection electrodes, respectively. The major challenge in 3D printing microchips for electrophoretic applications is the size of the microchannels, which should usually have cross‐sections well below 100 µm (due to the need for the dissipation of Joule heating). The required resolution is not easily possible with the common filament printers (fused deposition modelling (FDM)), and also the process may leave a porous substrate. Woolley and co‐workers have demonstrated that these problems can be overcome with stereolithographic printing, which employs a UV‐curing resin [78]. Microchannels with a cross‐section as small as 18 × 20 µm were achieved.

**FIGURE 5:**
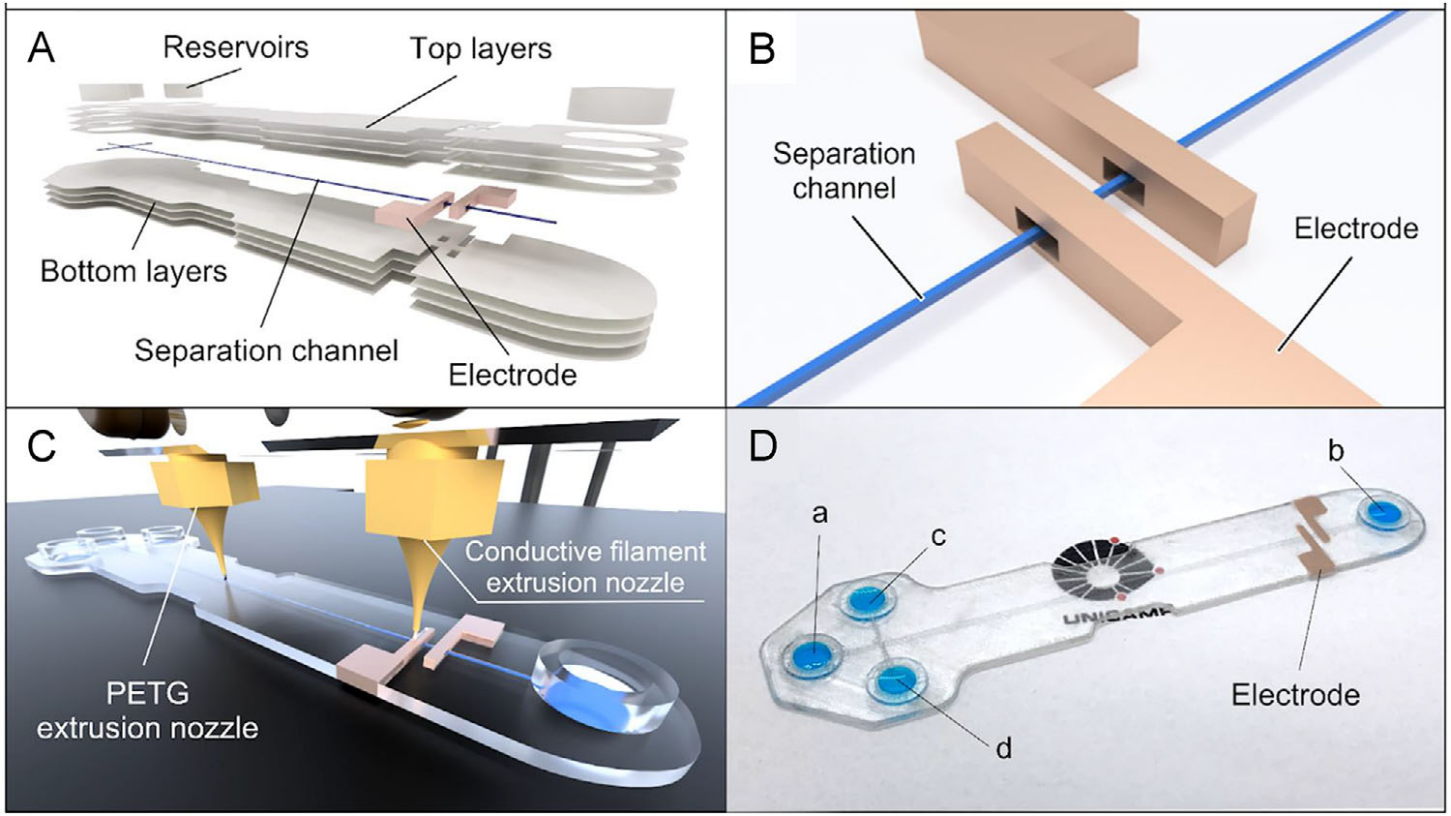
Microchip layers (A), electrodes (B), 3D printing process (C) and the final 3D‐printed microchip with C^4^D electrodes (D). *Source*: Reproduced from *Sensors & Actuators, B: Chemical* [77] with permission from Elsevier.

Until recently, most ME‐C^4^D devices used external planar electrodes, which were attached to the microchip surface or placed below the microchip. These electrodes usually compromised the C^4^D performance due to the relatively large distance from the separation channel and poor elimination of stray capacitance. With the advent of 3D printing, novel microchips were designed with electrodes wrapped around the separation channel [77, 79], emulating the concept of tubular electrodes in the CE‐C^4^D. Costa et al. created a device by stereolithographic printing with channels of 40 µm cross‐section and larger channels for electrodes. The latter were filled with molten gallium to form the electrodes and to establish electric contact with the C^4^D electronics [79]. Hong et al. [80] also reported a chip device with C^4^D electrodes created by filling channels with molten gallium, but the method used for producing the chip itself is not quite clear. Quero et al. employed an FDM technology with two separate nozzles for microchip printing, which consisted of a polymeric (polyethylene terephthalate glycol) body with injection/separation channels and electrodes made of three different conductive materials (polylactic acid (PLA)/graphene, PLA/carbon black and copper). The electrodes completely surrounded the 58 × 65 µm separation channel [77]. Although the electrodes were optimized on the basis of the design of tubular electrodes as used in CE‐C^4^D, no improvement in system sensitivity was achieved, possibly due to the lack of Faraday shielding. Figure 5 shows the different layers of the 3D‐printed device, electrode design, printing process, as well as the final microchip.

Wang et al. [81] reported an ME‐C^4^D device made from PMMA, which incorporated planar electrodes, including a grounded shield electrode to reduce stray capacitance. An inexpensive and simple approach to mass production of PMMA microfluidic devices was suggested by Coltro and co‐workers [82]. This consisted of the fabrication of a stiff PDMS master for soft embossing of the PMMA devices at ∼220°C–230°C and thermal sealing at 140°C.

Moore and co‐workers [83] developed a microfabricated planar device for electrochromatography. This features a separation channel made from silicon‐containing pillars, which act as a stationary phase. The C^4^D cell was made from glass and bonded to the silicon separation section. The separation of potassium and sodium ions and acetylsalicylate was demonstrated. However, the use of the semiconducting silicon is a complication as it requires the formation of a contiguous solid layer of SiO_2_ to prevent a bypass of the separation voltage. Chau et al. [84] studied the use of C^4^D in isoelectric focussing on a planar microfluidic device. In order to achieve detection, the separation channel was pressurized on conclusion of the focussing step to pass the analytes through the detector. Liang et al. [85] reported a C^4^D array based on 32 time‐multiplexed cells developed for direct monitoring in free‐flow electrophoresis.

### 2.4 Other Solution Analyses

Several authors have reported studies on C^4^D designs not intended for detection in CE but more general ionic conductivity measurements of solutions. Xiao and co‐workers studied the use of a C^4^D cell with five electrodes (three for excitation and two for signal pick‐up) [86] as well as of a dual cell, in which one was filled with a reference solution to compensate for a background conductivity [87]. Hantschke and Triantis [88, 89] carried out two modelling studies on a planar C^4^D with four electrodes in different geometrical arrangements. Takekawa et al. [90] reported a study of vapour‐depositing electrodes for a planar C^4^D. He et al. [91] modulated the frequency response of a circuitry for a planar C^4^D by adding an inductor and demonstrated its use at the relatively low frequencies of 50 and 29 kHz. Cao et al. [92] developed a device for testing the efficiency of desalting of biological samples.

## 3 Applications

### 3.1 Capillary Electrophoresis

C^4^D of analytes separated in standard capillaries (CE‐C^4^D) has been mainly used in the analyses of food, pharmaceutical, clinical and environmental samples and has followed the trends from previous years. Analyses of standard aqueous solutions have typically been used for the optimization of CE‐C^4^D systems, and less frequently, CE‐C^4^D has been also used for investigations of industrial samples. A complete list of CE‐C^4^D applications published in the reviewed period is given in Table 2 (sorted alphabetically by analyte name) and contains comprehensive information on the analytical methods, including analyte name(s), BGE solution composition, C^4^D parameters, CE separation mode, sample type and LOD(s). Additional information on recent applications of CE‐C^4^D can also be found in general review articles [31, 35] and review articles focussed on biomedical analysis [29, 32], food analysis [30] and amino acid/peptide analysis [28, 34].

**TABLE 2:** Applications of C^4^D in conventional capillary electrophore (CE).

Analytes	BGE composition	C^4^D parameters	Mode	Sample type	LODs	refs.
***Food analysis***						
Acesulfame‐K	n.r.	n.r.	LLE‐CZE FASS	Soy‐sauce	0.15 mg/kg	[97]
Alkali and alkaline earth metal cations	15 mM His, 50 mM acetic acid, 4 mM 18‐crown‐6, pH 4.0	eDAQ ER225, ET120; 100%, 100 kHz, gain: on	CZE modular	Honey	5–20 µM	[64]
Cadaverine, putrescine, spermine, spermidine	500 mM acetic acid, 180 mM 18‐crown‐6, pH 2.5	80 *V*_pp_, 550 kHz	EME‐CZE FASS	Tap and bottled water	0.06–6 µg/L	[96]
Fructose, glucose, lactose, sucrose	40 mM NaOH, pH 12.5	Admet; 44 *V*_pp_, 1.84 MHz	CZE	Beverages, honey, juice	≤1.7 mg/L	[166]
GHB, AHB, BHB	150 mM HEPES	20 *V*_pp_, 261 kHz	SPE‐CZE	Beverages	0.37 µM	[135]
Glucosamine, Ca^2+^	10 mM Tris adjusted to pH 5.0 with acetic acid	eDAQ ER815	CZE	Dietary supplements	0.05–1 mg/L	[167]
Glucosamine, Ca^2+^	10 mM Tris adjusted to pH 5.0 with acetic acid	eDAQ ER815	CZE	Nutrition samples	0.05–0.15 mg/L	[168]
Glyphosate, glufosinate, AMPA	1 mM His adjusted to pH 2.75 with acetic acid	eDAQ ER815	SPE‐CZE dual channel	Tea infusion	0.56–1.56 µg/L	[93]
Heavy metal cations	5 mM MES, 5 mM His, 3 mM HIBA, pH 5.0	eDAQ ER225, ET120; 100%, 100 kHz, gain: on	CZE modular	Honey	5–20 µM	[64]
Heavy metal cations	4.6 M acetic acid	40 *V*_pp_, 1 MHz, eDAQ ER225, ET120; 100 *V*_pp_, 800 kHz	CZE	Tap water	10 µg/L	[51]
Inorganic and organic anions	20 mM MES, 20 mM His, 30 µM CTAB	Admet; 50 *V*_pp_, 1.84 MHz	CZE	Tap water	n.r.	[56]
Inorganic and organic anions	40 mM MES, 40 mM His, pH 6.0	eDAQ ET120	CZE cap. coating	Beverages	0.36–24 mg/L	[169]
Inorganic anions	0.86 M acetic acid	C^5^D	CZE portable	Drinking water	1–4 mg/L	[65]
Inorganic anions	200 mM acetic acid or 12 mM His, 50 mM acetic acid, pH 4.1	40 *V*_pp_, 1 MHz eDAQ ER225, ET120; 100 *V*_pp_, 800 kHz	CZE	Tap water	8 µg/L	[51]
Inorganic anions	100 mM acetic acid	TraceDec; V: 0 dB, f: high, gain: 200%	CZE high‐speed	Drinking water, black tea, explosives	n.r.	[71]
Inorganic anions	400 mM Bis‐Tris, 400 mM MOPS, 2 mM 18‐crown‐6	TraceDec	CZE	Drinking water	3.0–6.8 µM	[70]
Inorganic anions	20 mM MES, 20 mM His, 2 mM 18‐crown‐6, 30 µM CTAB	Admet; 50 *V*_pp_, 1.84 MHz	CZE	Tap water	n.r.	[56]
Inorganic cations	15 mM His, 50 mM acetic acid, pH 4.0	eDAQ ER225, ET120; 100%, 100 kHz, gain: on	CZE modular	Honey	5–20 µM	[64]
Inorganic cations	0.86 M acetic acid	C^5^D	CZE portable	Drinking water	1–4 mg/L	[65]
Inorganic cations	20 mM MES, 20 mM His	40 *V*_pp_, 1 MHz, eDAQ ER225, ET120; 100 *V*_pp_, 800 kHz	CZE	Tap water	20 µg/L	[51]
Inorganic cations	300 mM Bis‐Tris, 300 mM MOPS, 2 mM 18‐crown‐6	TraceDec; V:—6 dB, f: 3× high, gain: 200%	CZE high‐speed	Drinking water, black tea, explosives	2.5–5.2 µM	[71]
Inorganic cations	400 mM Bis‐Tris, 400 mM MOPS, 2 mM 18‐crown‐6	TraceDec	CZE	Drinking water	2.1–3.6 µM	[70]
Inorganic cations	20 mM MES, 20 mM His, 2 mM 18‐crown‐6, 30 µM CTAB	Admet; 50 *V*_pp_, 1.84 MHz	CZE	Tap water	n.r.	[56]
Inorganic cations	12 mM His, 1 M acetic acid, 2 mM 18‐crown‐6	eDAQ ER225	CZE	Milk	0.05–0.1 mg/L	[95]
MPPA	30 mM His adjusted to pH 6.7 with MOPS, 10 µM CTAB	eDAQ ER815	SPE‐CZE dual channel	Tea infusion	0.54 µg/L	[93]
Orthophosphate, pyrophosphate, tripolyphosphate	0.5 M acetic acid, pH 2.5	3 *V*_pp_, 530 kHz	CZE	Tap water, instant noodles	0.41–0.58 mg/L	[170]
Scopolamine, butylscopolamine	20 mM butyric acid, 7 mM NaOH, pH 4.5	4 *V*_pp_, 1.1 MHz	CZE	Beverages	2.4–2.8 µM	[101]
Vitamins B_1_, B_5_, B_9_	12 mM Arg adjusted to pH 7.5 with acetic acid, 10% acetonitrile	eDAQ ER815	CZE	Nutrition samples	0.3–0.6 mg/L	[168]
Vitamin B_6_, Ca^2+^	10 mM Arg adjusted to pH 5.0 with acetic acid, 20% acetonitrile	eDAQ ER815	CZE	Nutrition samples	0.1–1 mg/L	[171]
Vitamin B_6_, Ca^2+^	10 mM Arg adjusted to pH 5.0 with acetic acid, 20% acetonitrile	eDAQ ER815	CZE	Nutrition samples	0.05–1 mg/L	[168]
Pharmaceutical, clinical and other complex sample analysis						
Adenosine, cordycepin	0.25 M acetic acid	eDAQ ER815	CZE dual channel	Chinese medicine	11.2–11.6 µg/g	[109]
Amikacin, streptomycin, kanamycin A, kanamycin B	200 mM acetic acid	eDAQ ER815; 90%, 500 kHz	CZE	Pharmaceuticals	0.5–3 mg/L	[99]
Amino acids	0.5 M acetic acid, 40 mM α‐CD, 0.1% HEC 0.5 M acetic acid, 20 mM α‐CD, 5 mM β‐CD, 0.1% HEC	TraceDec; V: 50%, f: High, gain: 50%	CZE	Cell culture supernatant	10–50 µM	[104]
Amino acids	400 mM acetic acid, 0.1% (v/v) Tween 20, pH 2.1	Admet; 50 *V*_pp_, 1.84 MHz	CZE	DBS	4–6 µM in blood	[121]
Amino acids	3.2 M acetic acid	WynSep	CZE	Mouse plasma	2 µM	[110]
Amino acids	8.5 M acetic acid, pH 1.37	Admet; 80 *V*_pp_, 1 MHz	MD‐CZE	Blood, plasma, tears	0.12–0.28 µM	[112]
Amino acids	3.2 M acetic acid, 13% (v/v) methanol	Admet; 80 *V*_pp_, 1 MHz	CZE	Blood plasma	0.7–0.9 µM	[111]
Amino acids, nucleosides, short peptides	2 M acetic acid	eDAQ ER815, ET120; 100%, 1000 kHz	CZE	Cell culture media	5 µM	[172]
Amoxicillin	0.5 M acetic acid	Admet; 80 *V*_pp_, 1 MHz	MD‐CZE LVSS cap. coating	Serum, tissue dialysate	143, 148 ng/mL LOQ	[117]
Antibiotics	50 mM formic acid	eDAQ ER225, ET120; 80%, 200 kHz	CZE	Pharmaceuticals	0.5–5.88 mg/L	[100]
Arginine, ibuprofen	10 mM CHES, 10 mM boric acid adjusted to pH 8.4 with KOH	4 *V*_pp_, 1.1 MHz	CZE	Pharmaceuticals	5.3–10.0 µM	[103]
Atropine, scopolamine	0.5 M acetic acid, 0.25% (w/v) β‐CD	eDAQ ER225, ET120; 60%, 300 kHz, gain: on	CZE portable	Plant extracts, pharmaceuticals	0.5–1.5 mg/L	[107]
Butyrate	10 mM Tris, 10 mM MOPS, pH 6.6	TraceDec; V: 0 dB, f: medium, gain: 100%	CZE online hydrolysis	Plant extracts	0.5 µM	[173]
Ceftazidime	3.2 M acetic acid, 13% (v/v) methanol	Admet; 80 *V*_pp_, 1 MHz	MD‐CZE LVSS cap. coating	Serum, tissue dialysate	318, 339 ng/mL LOQ	[117]
Creatinine	50 mM acetic acid, 0.1% (v/v) Tween 20, pH 3.0	Admet; 50 *V*_pp_, 1.84 MHz	CZE	DBS	5 µM in blood	[122]
Creatinine	LE: 10 mM NH_4_OH, 20 mM MES, pH 6.25 TE: 10 mM EACA, 5 mM acetic acid, pH 4.8	Villa Labeco	c‐ITP	Urine	3 µM	[139]
Creatine, 2‐aminobutyric acid, acetyl‐carnitine, amino acids	8.5 M acetic acid, pH 1.37	Admet; 80 *V*_pp_, 1 MHz	CZE cap. coating, LVSS	Blood plasma	0.2–0.4 µM	[118]
Cysteine, homocysteine, glutathione, methionine	4 M acetic acid, pH 2.08	90 *V*_pp_, 750 kHz	SPE‐CZE LVSS	Saliva	0.15–1.5 ng/mL	[128]
Dimethylarginine	0.75 M acetic acid, pH 2.45	Admet; 80 *V*_pp_, 1 MHz	CZE cap. coating whole cap. injection	Blood plasma	16–22 nM	[113]
GHB, AHB, BHB	150 mM HEPES	20 *V*_pp_, 261 kHz	SPE‐CZE	Urine	0.37 µM	[135]
Hydrochlorothiazide, losartan, K^+^	10 mM boric acid adjusted to pH 9.0 with NaOH	4 *V*_pp_, 1.1 MHz	CZE	Pharmaceuticals	3–10 µM	[102]
3‐Hydroxybutyrate	80 mM MES, 80 mM His	Admet; 80 *V*_pp_, 1 MHz	CZE cap. coating, LVSS	Blood serum	0.43 µM	[115]
d,l‐2‐Hydroxyglutaric acid	50 mM MOBS, 0.001% (w/v) polybrene, 30 mM vancomycin	TraceDec; V: −18 dB, f: high, gain: 100%	CZE chiral	Urine	0.497, 0.567 mg/L	[134]
Ibotenic acid, muscimol	1 M formic acid, pH 1.88	n.r.	CZE	Plant extracts	n.r.	[108]
Inorganic anions	10 mM MES, 10 mM Arg, pH 7.5	TraceDec	CZE cap. coating	Saliva	15.2–39.3 µM	[127]
Inorganic anions	20 mM MES, 20 mM His, 2 mM 18‐crown‐6, 30 µM CTAB, pH 6	Admet; 50 *V*_pp_, 1.84 MHz	CZE	Sweat	n.r.	[130]
Inorganic anions	20 mM MES, 20 mM His, 2 mM 18‐crown‐6, 30 µM CTAB	Admet; 50 *V*_pp_, 1.84 MHz	CZE	Sweat	n.r.	[56]
Inorganic anions	20 mM MES, 20 mM His, 30 µM CTAB	Admet; 50 *V*_pp_, 1.84 MHz	CZE	Sweat	n.r.	[66]
Inorganic anions	20 mM MES, 20 mM His, 2 mM 18‐crown‐6, 30 µM CTAB, pH 6.0	Admet; 50 *V*_pp_, 1.84 MHz	CZE	Sweat	n.r.	[129]
Inorganic cations	10 mM MES, 5 mM Arg, 1.4 mM 18‐crown‐6, pH 6.2	TraceDec	CZE cap. coating	Saliva	0.5–5.3 µM	[127]
Inorganic cations	1.6 M acetic acid, 0.1% (v/v) Tween 20, 1 mM 18‐crown‐6, pH 2.1	Admet; 50 *V*_pp_, 1.84 MHz	SI‐CZE	DBS	Li^+^: 0.4 mg/L in blood	[124]
Inorganic cations	20 mM MES, 20 mM His, 2 mM 18‐crown‐6, 30 µM CTAB, pH 6	Admet; 50 *V*_pp_, 1.84 MHz	CZE	Sweat	n.r.	[130]
Inorganic cations	20 mM MES, 20 mM His, 2 mM 18‐crown‐6, 30 µM CTAB	Admet; 50 *V*_pp_, 1.84 MHz	CZE	Sweat	n.r.	[56]
Inorganic cations	20 mM MES, 20 mM His, 30 µM CTAB	Admet; 50 *V*_pp_, 1.84 MHz	CZE	Sweat	n.r.	[66]
Inorganic cations	20 mM MES, 20 mM His, 2 mM 18‐crown‐6, 30 µM CTAB, pH 6.0	Admet; 50 *V*_pp_, 1.84 MHz	CZE	Sweat	n.r.	[129]
Inorganic cations and creatinine	1 M acetic acid, 1 mM 18‐crown‐6, 20% (v/v) methanol, pH 2.5	Admet; 50 *V*_pp_, 1.84 MHz	CZE	DUS	n.r.	[125]
Inorganic cations, inorganic anions, organic anions	20 mM MES, 20 mM His, 2 mM 18‐crown‐6, 30 µM CTAB	Admet; 50 *V*_pp_, 1.84 MHz	CZE	EBC	n.r.	[126]
Inorganic, monocarboxylic, dicarboxylic and tricarboxylic anions	50 mM TEA, 55 mM acetic acid, 5% glycerol, pH 5.5	eDAQ; 100%, 800 kHz, 900 kHz, gain: on	CZE	Cell culture media	1–25 µM	[146]
Inosine	150 mM Tris adjusted to pH 8.5 with formic acid	eDAQ ER815	CZE dual channel	Chinese medicine	22 µg/g	[109]
Lactate	40 mM MES, 40 mM His, 0.05 mM CTAB, 0.1 mM TRIME‐β‐CD, pH 6.0	eDAQ ET120	CZE	Sweat	42 µM	[131]
Lactate	10 mM MES, 10 mM His, 0.2 mM CTAB	3 *V*_pp_, 510 kHz	CZE	Sweat	3.1 µM	[132]
Lactate	10 mM His, 15 mM glutamic acid, 30 µM CTAB, pH 4.6	Admet; 50 *V*_pp_, 1.84 MHz	CZE	Blood plasma	6 µM	[114]
Lactate, pyruvate	30 mM CHES, 30 mM Tris, 0.025% PEI, pH 8.4	TraceDec; V: 18 dB, f: 2× high, gain: 200%	SI‐CZE	Media for cell cultivation	8–17 nM	[106]
Lactate, pyruvate	30 mM CHES, 30 mM Tris, 0.025% PEI, pH 8.4	TraceDec; V: 18 dB, f: 2× high, gain: 200%	CZE	Media for cell cultivation	10–20 nM	[105]
Lidocaine	50 mM MES, 50 mM CAP, pH 5.2	Grey Scan ETD‐100	CZE portable	Pharma production contamination	0.13 µg/swab	[98]
Memantine, amantadine, rimantadine	1.5 M acetic acid, 0.05 mM β‐CD, pH 2.5	eDAQ ER125; 2 *V*_pp_, 500 kHz	DLLME‐CZE FASS	Serum, urine	0.9–1.2 nM	[137]
Methadone	3 M acetic acid	Admet; 80 *V*_pp_, 1.8 MHz	EME‐CZE	Urine, serum	0.21 mg/L	[136]
Neomycin, tobramycin, polymyxin	200 mM acetic acid	eDAQ ER815; 90%, 500 kHz	CZE	Pharmaceuticals	0.5–3 mg/L	[99]
Organic anions	10 mM MES, 10 mM His, 1% β‐CD, 1% polybrene	eDAQ; 100%, 200 kHz, gain: on	CZE cap. coating	Faeces	0.1–0.89 mg/L	[68]
Orotic acid	20 mM MES, 20 mM His, 0.1 mM CTAB, pH 6.5	TraceDec; V: −12 dB, f: n.r., gain: 100%	CZE FASS	Urine	0.014 mg/L	[133]
Phenothiazine derivatives	30 mM MES, 30 mM aspartic acid, 4 mM HP‐γ‐CD, pH 2.5	eDAQ ER225; 2 *V*_pp_, 400 kHz	DLLME‐CZE FASS chiral	Urine, serum	0.24–0.28 nM	[138]
Scopolamine, butylscopolamine	20 mM butyric acid, 7 mM NaOH, pH 4.5	4 *V*_pp_, 1.1 MHz	CZE	Pharmaceuticals	2.4–2.8 µM	[101]
Uric acid	30 mM MES, 30 mM His, 30 µM CTAB	Admet; 50 *V*_pp_, 1.84 MHz	CZE	DBS	n.r.	[123]
Vancomycin, teicoplanin	15 mM Arg, 15 mM acetic acid, pH 9.0	eDAQ ER815; 90%, 500 kHz	CZE	Pharmaceuticals	0.5–3 mg/L	[99]
Vigabatrin, pregabalin, gabapentin	0.5 M acetic acid	Admet; 80 *V*_pp_, 1 MHz	CZE cap. coating, LVSS	Blood serum	18.3–22.8 nM	[116]
Vitamin B_6_, Ca^2+^	10 mM Arg adjusted to pH 5 with acetic acid, 20% acetonitrile	eDAQ ER815	CZE	Pharmaceuticals	0.1–1 mg/L	[171]
Environmental analysis						
Alkyl sulphate surfactants (C8, C10, C12, C14)	50 mM Tris, 20 mM His, pH 8.95	3S Analysis, eDAQ, ER815	SPE‐CZE portable	Waste water	2.08–3.16 mg/L	[149]
ClO_3_^−^, NO_3_^−^, ClO_4_^−^	10 mM His, 100 mM acetic acid, pH 3.69	TraceDec; V: 0 dB, f: 2× high, gain: 200%	GEMBE	Explosive powders	1.8–2.0 µM	[148]
Diethylamine, triethylamine	0.5 M acetic acid	AD7745	CZE portable	Air, gas analysis	0.050–0.054 µM LOQ	[153]
Inorganic and organic anions	Grey Innovation	Grey Scan ETD‐100	CZE	Surface wipes	n.r.	[147]
Inorganic anions	12 mM His adjusted to pH 4.0 with acetic acid	n.r.	CZE	Air samples	0.02 mg/L	[154]
Inorganic anions, nitrite, nitrate	20 mM Tris, 210 mM acetic acid, pH 3.7	TraceDec; V: n.r., f: high, gain: 200%	CZE	Lake water	13 µM, 0.18 mg N/L	[151]
Inorganic cations	Tris, EDTA, acetic acid, PVP (concentrations n.r.)	n.r.	CZE portable	Soil samples	n.r.	[67]
Inorganic cations	12 mM His, 2 mM 18‐crown‐6 adjusted to pH 4.0 with acetic acid	n.r.	CZE	Air samples	n.r.	[154]
Inorganic cations, amines, BSA, organic acids, nerve agent degradation products	0.5 M acetic acid	AD7745	CZE portable	Air samples	n.r.	[72]
Inorganic cations, amino acids	5 M acetic acid	3.3 *V*_pp_, 32 kHz, square wave	CZE portable	Sea water	≤5.2 µM LOQ	[74]
Inorganic, monocarboxylic, dicarboxylic and tricarboxylic anions	50 mM TEA, 55 mM acetic acid, 5% glycerol, pH 5.5	eDAQ; 100%, 800 kHz, 900 kHz, gain: on	CZE	Lake water	1–25 µM	[146]
K^+^	n.r.	n.r.	CZE	Soil samples	n.r.	[152]
Nitrate	MES, His (concentrations n.r.)	n.r.	CZE portable	Soil samples	n.r.	[67]
Nonsteroidal anti‐inflammatory drugs (Ibu, Ket, ASA, Dic)	20 mM His, 15 mM Tris, 2 mM HP‐β‐CD, 10% (v/v) methanol, pH 8.6	TraceDec	SPE‐CZE	Waste water	0.062–0.5 mg/L	[174]
Paracetamol, *p*‐aminophenol	20 mM β‐alanine adjusted to pH 11 with NaOH, 14% (v/v) methanol	TraceDec	SPE‐CZE	Waste water	0.3125 mg/L	[175]
Perfluoroheptanoate, perfluorooctanoate, perfluorooctanesulfonate	0.75 mM MOPS, 1.5 mM TEA, acetonitrile/methanol 50/50% (v/v)	TraceDec	NACE	Waste water	0.3–0.75 µM	[150]
Phosphate	26 mM acetic acid	n.r.	CZE portable	Soil samples	n.r.	[67]
Industrial applications						
Dodecyldimethylbenzylammonium bromide, dodecyltrimethylammonium bromide	25 mM lactic acid, 25 mM β‐alanine	eDAQ, ET120	CE portable smartphone	Disinfector analysis	n.r.	[69]
K^+^, glyphosate	20 mM MES, 20 mM His, pH 6.1	eDAQ ER225, open C^4^D, 24 *V*_pp_, 32 kHz, square wave	CZE	Roundup PowerFlex herbicide	10 µM	[46]
Monosaccharides	50 mM NaOH, 22 mM disodium phosphate, 0.2 mM CTAB, pH 12.4	Admet; 44 *V*_pp_, 1.84 MHz	CZE	Bovine fetuin	5–7 mg/L	[176]
Orthophosphate, pyrophosphate, tripolyphosphate	0.5 M acetic acid, pH 2.5	3 *V*_pp_, 530 kHz	CZE	Toothpaste, mouthwash, antiseptic oral spray	0.41–0.58 mg/L	[170]
Standard solutions						
Alkyl sulphates (C8, C10, C12, C14)	50 mM Tris, 20 mM His, pH 8.0	3S Analysis	SPE‐CZE	Standard solutions	5–10 µM	[177]
Amines	500 mM acetic acid, pH 2.5	Open C^4^D	CZE	Standard solutions	n.r.	[62]
Amino acids and inorganic cations	5 M acetic acid	eDAQ ER815, ET120; 100%, 1200 kHz, gain: on	CZE	Standard solutions	n.r.	[76]
AMP, ADP, benzoate, gluconate, citrate, dichromate	70 mM Tris, 70 mM CHES, 0.2 mM CTAB	77 kHz, 157 kHz	CZE	Standard solutions	0.1–0.3 µM	[59]
(Ferrocenylmethyl)trimethylammonium iodide	10 mM ammonium acetate, 1 M acetic acid in acetonitrile	4 *V*_pp_, 1.1 MHz	NACE	Standard solutions	n.r.	[155]
Glyphosate and its metabolites	Various BGE solutions, pH 1.25–12.02	eDAQ ER225, ET120; 100%, 300 kHz, gain: on	CZE	Standard solutions	n.r.	[163]
Inorganic anions	200 mM acetic acid, pH 2.6	40 *V*_pp_, 1.2 MHz, sine wave	CZE	Standard solutions	6–7 nM	[55]
Inorganic anions, organic anions, ADP, GMP, AMP	2 mM Tris, 70 mM acetic acid, pH 3.3 or 70 mM Tris, 70 mM CHES, 0.2 mM CTAB	157 kHz	CZE, cap. coating	Standard solutions	n.r.	[61]
Inorganic cations	20 mM MES, 20 mM His	40 *V*_pp_, 1.2 MHz, sine wave	CZE	Standard solutions	9–24 nM	[55]
Inorganic cations	100 mM acetic acid, 20 mM LiOH, pH 4.1	Admet	CZE	Standard solutions	n.r.	[161]
Inorganic cations	50 mM formic acid, pH 2.5	Open C^4^D	CZE	Standard solutions	n.r.	[62]
Inorganic cations	20 mM Bis‐Tris, 20 mM MOPS, 3.5 mM 18‐crown‐6	77 kHz, 157 kHz	CZE	Standard solutions	8–12 µM	[59]
Inorganic cations, amino acids	10 mM Bis‐Tris, 10 mM MOPS	TraceDec; f: 77 kHz, 157 kHz	CZE	Standard solutions	2–2.6 µM	[60]
Inorganic cations, amino acids	5 M acetic acid	20 *V*_pp_, 800 kHz; eDAQ ER815; 100%, 1200 kHz	CZE	Standard solutions	n.r.	[57]
Inorganic cations, amino acids	5 M acetic acid	eDAQ ER225; 100%, 1200 kHz	CZE	Standard solutions	n.r.	[75]
Inorganic cations, amino acids	5 M acetic acid	eDAQ ER815; 90%, 1200 kHz	CZE automated	Standard solutions	n.r.	[73]
Inorganic cations, amino acids	40 mM Tris, 40 mM CHES	157 kHz	CZE	Standard solutions	n.r.	[61]
Inorganic cations, heavy metal cations	5 M acetic acid or 0.5 M acetic acid	eDAQ	CZE	Standard solutions	30–75 µg/L	[162]
Inorganic cations, organic cations, anions	0.5 M acetic acid, pH 2.53	3.3 *V*_pp_, 32 kHz, square wave	CZE	Standard solutions	n.r.	[160]
K^+^, Na^+^, Co^2+^, Zn^2+^	50 mM Tris, 50 mM TAPS, 0.1 mM PAR, pH 8.2	157 kHz	CZE portable	Standard solutions	1.2–21 µM	[58]
K^+^, Na^+^, Li^+^	30 mM MES, 30 mM His, pH 6.1 or 500 mM acetic acid	Open C^4^D	CZE	Standard solutions	n.r.	[158]
K^+^, Na^+^, Li^+^	500 mM acetic acid	4 *V*_pp_, 1.1 MHz	CZE	Standard solutions	n.r.	[159]
K^+^, Na^+^, Li^+^, TrisH^+^	20 mM MES, 20 mM His, pH 6.1	eDAQ ER225, open C^4^D, 24 *V*_pp_, 32 kHz, square wave	CZE	Standard solutions	4–12 µM	[46]
Mixed micelles	10 mM borate and PEO	TraceDec	MEKC	Standard solutions	n.r.	[164]
Organic acids	30 mM MES, 30 mM His, pH 6.1 10 mM NaHCO_3_, pH 8.3	Open C^4^D	CZE	Standard solutions	n.r.	[62]
Organic acids	50 mM MES, 5 mM HTAOH, pH 5.5	eDAQ ER815	CZE	Standard solutions	n.r.	[178]
Organic acids	5 mM benzoate, 0.2 mM CTAB adjusted to pH 8.0 with Tris	77 kHz, 157 kHz	CZE	Standard solutions	0.2–0.5 µM	[59]
Polymers	Various BGE solutions	TraceDec; V: 0 dB, f: (M, H, 2× H), gain: 50%	CZE TDA	Standard solutions	n.r.	[165]
Polymers	10 mM TBAH in NMP	TraceDec	NACE	Standard solutions	n.r.	[156]
Proteins	Catholyte: 35 mM NaOH, 2% HEC; anolyte: 35 mM H_3_PO_4_, 2% HEC	eDAQ ER225, ET121; 200 *V*_pp_, 300 kHz	µCIEF	Standard solutions	10 nM	[84]
Proteins	n.r.	n.r.	FFE	Standard solutions	n.r.	[85]
Rare earth metals	20 mM Arg, 3 or 10 mM HIBA adjusted to pH 4.2 with acetic acid	40 *V*_pp_, 1.2 MHz, sine wave	CZE	Standard solutions	29–410 nM	[55]
Salicylic acid	10 mM EPPS adjusted to pH 7.4 with LiOH	Admet; 50 *V*_pp_, 1.0 MHz	CE frontal analysis	Standard solutions	1.65 µM	[157]

Abbreviations: µCIEF, microfluidic capillary isoelectric focussing; ADP, adenosine diphosphate; AHB, α‐hydroxybutyric acid; AMP, adenosine monophosphate; AMPA, aminomethylphosphonic acid; Arg, l‐arginine; BHB, β‐hydroxybutyric acid; Bis‐Tris, bis‐(2‐hydroxyethyl)‐imino‐tris‐(hydroxymethyl)‐methane; C^5^D, compensated capacitively coupled contactless conductivity detection; CAP, α‐aminocaproic acid; c‐ITP, capillary isotachophoresis; DBS, dried blood spot; DLLME, dispersive liquid‐liquid microextraction; DUS, dried urine spot; EACA, ε‐aminocaproic acid; EBC, exhaled breath condensate; EME, electromembrane extraction; EPPS, 4‐(2‐hydroxyethyl)piperazine‐1‐propanesulfonic acid; FFE, free‐flow electrophoresis; GEMBE, gradient elution moving boundary electrophoresis; GHB, γ‐hydroxybutyric acid; HEC, 2‐hydroxyethyl cellulose; HIBA, α‐hydroxyisobutyric acid; His, l‐histidine; HP‐β‐CD, hydroxypropyl‐β‐cyclodextrin; HP‐γ‐CD, hydroxypropyl‐γ‐cyclodextrin; HTAOH, hexadecyltrimethylammonium hydroxide; LLE, liquid–liquid extraction; MD, microdialysis; MES, 2‐(*N*‐morpholino)‐ethanesulfonic acid; MOBS, 4‐(*N*‐morpholino)‐butanesulfonic acid; MPPA, 3‐(methylphosphonico)propionic acid; n.r., not reported; NMP, *N*‐methyl‐2‐pyrrolidone; PAR, 4‐(2‐pyridylazo)resorcinol; PEI, poly(ethyleneimine); PEO, poly(ethylene oxide); SPE, solid phase extraction; TBAH, tetrabutylammonium hydroxide; TDA, Taylor dispersion analysis; TEA, triethylamine; TRIME‐β‐CD, heptakis (2,3,6‐tri‐*O*‐benzoyl)‐β‐cyclodextrin; Tween 20, polyethylene glycol sorbitan monolaurate.

#### 3.1.1 Food Samples

Analysis of food products and the determination of herbicides therein need to consider the presence of their decomposition products, which may have similar but also significantly different physical–chemical properties as the parent compound(s). As CE is preferably used to determine alike compounds in one analytical run, the simultaneous determination of structurally different components might be challenging. The major metabolites of glyphosate and glufosinate are aminomethylphosphonic and 3‐methylphosphonico propionic acid, respectively, with different structures and different requirements on CE separation conditions. Two BGE solutions with significantly different pH values (2.75 and 6.7) were found optimal for the determination of the two acids [93]. The analysis of all four compounds using a standard one‐capillary CE instrument would, thus, be tedious and time‐consuming because two different BGE solutions have to be used and the CE system cleaned before the BGE exchange. Nguyen et al. [93] therefore adopted the multichannel CE approach [94], which enables the determination of diverse analytes in separate capillaries using different BGE solutions. The use of parallel separation channels is, in contrast to HPLC, readily possible in CE‐C^4^D because of the low cost of the capillaries, the high‐voltage modules and the detector. Improved sample throughput and good separation of all four analytes in tea infusions were achieved using a CE‐C^4^D with two independent separation channels.

Analytes in real samples may not only have different electrophoretic properties, but they may also differ in their amenability to various detection schemes. Milk contains high concentrations of inorganic cations and whey proteins, and although the former are easily detected in diluted milk by C^4^D, C^4^D does not offer sufficient sensitivity for the determination of the latter. Proteins, however, can generally be detected with sufficient sensitivity in the UV region, and the same capillary can be employed for the determination of both groups of analytes when used with two (or even more) detectors. Zhao et al. have demonstrated simultaneous C^4^D and UV–Vis detection of the two groups of analytes in milk samples after various heat‐treatment processes and observed significant differences in protein contents for specific processes [95].

The modular CE‐C^4^D instrument designed by Furter et al. [64] was used for the determination of inorganic and heavy metal cations in honey samples (diluted with deionized (DI) water and filtered only), reporting the presence of all major inorganic cations and trace amounts of zinc. Zhang and co‐workers demonstrated their high‐speed CE‐C^4^D instrument for rapid separations (in ≤22 s) of inorganic cations and anions in drinking water samples and black tea extracts [71].

**FIGURE 6:**
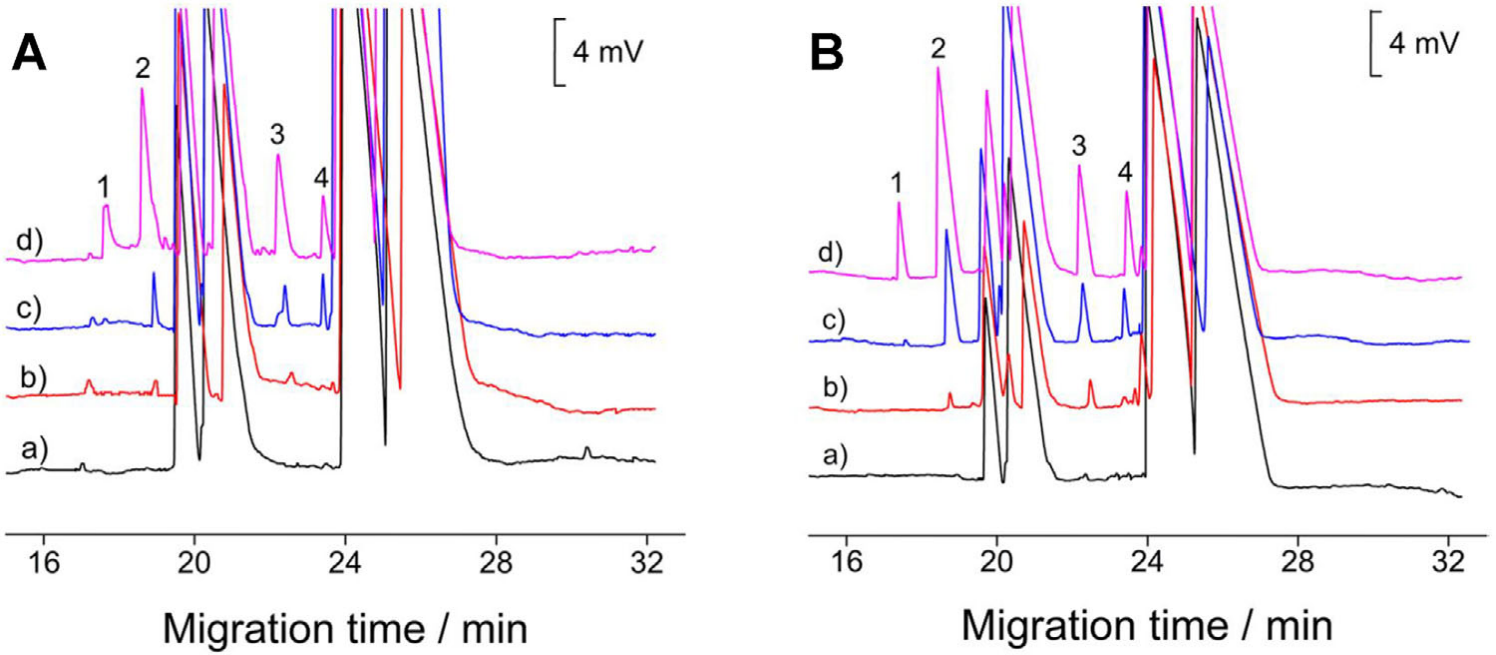
Typical sample electropherograms of tap water (A) and bottled drinking water (B) non‐spiked (a) and spiked (b—d) with different concentrations of spermine (1), spermidine (2), putrescine (3) and cadaverine (4). *Source*: Reproduced from *Food Analytical Methods* [96] with permission from Springer.

The determination of some analytes in food products requires only a simple dilute‐and‐shoot approach as their concentrations are high and matrix components are diluted to concentrations, which are not deleterious to the CE performance; these are typically the afore‐mentioned inorganic ions and proteins. On the other hand, many analytes are present in much smaller quantities in foods, and although the sensitivity of CE detection techniques (including C^4^D) might be sufficient for their determination in clean samples, the presence of the sample matrix makes it not possible. These are, for example, biogenic amines, which are present in foods in much smaller concentrations than inorganic cations but have similar electrophoretic properties. Food samples have to be, therefore, pretreated before their CE analyses to increase the concentration of amines and suppress the effect of matrix cations on their separation, which can be achieved, for example, by electromembrane extraction (EME). A method for the determination of four major biogenic amines (cadaverine, putrescine, spermine and spermidine) in bottled and drinking water has been developed on the basis of an off‐line EME and in‐line field amplified sample stacking (FASS) [96], resulting in a maximum enrichment factor of 1853 and LOD at the 0.06 µg/L level; see Figure 6. A CE method with FASS preconcentration was also presented for the determination of acesulfame‐K in soy sauce [97].

#### 3.1.2 Pharmaceutical, Clinical and Other Complex Samples

The pharmaceutical industry requires fast, simple, automated and reliable analytical methods for the quality control in drug production. CE‐C^4^D fulfils all these requirements and offers a perfect solution for many pharmaceutical applications. Drugs and pharmaceutical formulations are typically charged species and are thus amenable to electrophoretic separations on the basis of the charge and size of the analytes. They are also amenable to the conductivity detection, which monitors the differences in conductivities between the analyte zone and the BGE solution. Moreover, CE‐C^4^D is perfectly suited for portability and on‐site measurements, which are desirable in many pharmaceutical applications, too.

Breadmore and co‐workers have shown that the commercial portable CE‐C^4^D system (Grey Scan ETD‐100) can be used for the validation of cleaning processes in pharmaceutical facilities [98]. Lidocaine was selected as a model drug, and swab‐based sampling was carried out to determine the residual contamination of the production equipment. The collection swabs were used for an automated extraction and on‐site analysis by the Grey Scan system, which proved sufficient specificity and sensitivity for the identification of lidocaine and other contaminant drugs in about 60 s.

Even more important is the purity of the drug, and a proper quantification of active substances is crucial for the pharmaceutical industry. Expensive instrumentation (e.g., HPLC–MS/MS) is usually applied for drug quality control, which, however, is not affordable for many laboratories. Moreover, some drugs are not suited for standard detection techniques (UV–Vis or MS) due to their physical–chemical properties, and their quantification might be compromised. A lab‐made CE‐C^4^D was, therefore, used for the quality control of pharmaceutical products as an affordable alternative suitable for rapid and sensitive determination of three groups of aminoglycoside and glycopeptide antibiotics [99]. A CE‐C^4^D method has been optimized for the simultaneous determination of 12 different antibiotics (aminoglycosides, fluoroquinolones, tetracyclines and macrolides) in pharmaceutical products using an Agilent CE instrument with a dual detection feature [100]. C^4^D was shown to be superior to UV–Vis detection, which was able to detect only 6 of the 12 analytes; moreover, C^4^D achieved comparable or higher sensitivities.

“Time is money” and it particularly applies to the pharmaceutical analysis. CE offers several tools for speeding up the analysis by using short separation capillaries, short effective capillary lengths, high separation voltages or the short‐end injection mode in commercial CEs. A combination of all or some of these tools can significantly increase the sample throughput. Fast CE analyses are, thus, highly attractive for pharmaceutical laboratories as reducing the analysis times consequently reduces also the analysis costs (in addition to minimum costs of consumables and reagents, which CE offers). CE‐C^4^D was used by Richter and co‐workers for rapid analyses of various drugs and their counter‐ions in pharmaceutical formulations. Scopolamine and butylscopolamine were determined in about 120 s [101], potassium, hydrochlorothiazide and losartan in about 40 s [102], and arginine and ibuprofen in about 35 s [103] using uncoated fused silica (FS) separation capillaries with total and effective lengths of 50 and 10 cm, respectively. Despite the short separation times, it should be noted that capillary flushing, conditioning and sample injection have to be added to the total analysis time. The real sample throughput can, thus, reach approximately 20–30 samples/hour for CE‐C^4^D, which, in comparison to other separation methods, is still exceptional.

Cell culture supernatants are rather complex mixtures consisting of high concentrations of salts, buffers, saccharides and vitamins and low concentrations of target analytes. Their analyses are important as they provide information about the viability of the cultured cells and are typically done by HPLC. CE‐C^4^D was shown as an alternative technique for the determination of amino acids [104] and lactate/pyruvate [105] in these media. An additional step towards full automation of the monitoring process was demonstrated by Alhusban et al. [106] by the coupling of sequential injection (SI) to the CE‐C^4^D. Thus, the supernatant was automatically transferred from the culture flask to the SI‐CE interface for regular sampling (every 50 min), and the concentrations of lactate/pyruvate were continuously monitored for 72 h. A significant increase in their concentrations was observed for treated versus untreated control cells and was correlated with the expression of monocarboxylate transporter genes. CE‐C^4^D was also used for the analysis of other pharma‐related samples, such as plant extracts and extracts of traditional Chinese medicines. These included the determination of atropine and scopolamine [107], ibotenic acid and muscimol [108], and adenosine, cordycepin and inosine [109].

Most applications of CE‐C^4^D have been reported for the analyses of human body fluids. Body fluids are important samples in clinical, toxicological and forensic analysis, with blood (in the form of plasma and serum) being the golden standard. Blood samples contain numerous matrix components, which usually interfere with standard analytical methods, including CE‐C^4^D. A proper sample treatment is, therefore, necessary before their analyses. Due to the relatively high tolerance of CE separation techniques and equipment to many low‐molecular‐mass matrix components, blood plasma/serum can be injected after precipitation of high‐molecular‐mass compounds and centrifugation of the precipitate. The procedure involves only addition of acetonitrile or methanol to the sample, is rather quick and requires little sample volume. As the dilution is not significant (usually two‐ to threefold), it can be used for the determination of high‐ as well as low‐abundant analytes. A typical example of this approach is the determination of amino acids, which is important in many clinical applications and CE‐C^4^D offers several benefits in comparison to other analytical techniques: (i) direct detection with no need for derivatization, (ii) sufficient sensitivity for endogenous amino acid concentrations and (iii) comparable performance for all target analytes are the major advantages, which have been demonstrated in multiple contributions [110, 111, 112, 113].

**FIGURE 7:**
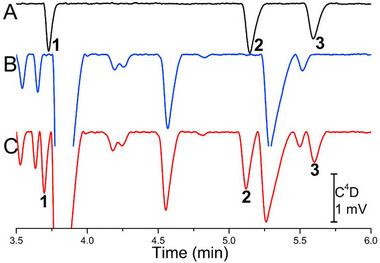
Electropherograms of human serum in a coated FS capillary, (A) standard solution at a level of 0.1 µg/mL vigabatrin (1), pregabalin (2) and gabapentin (3); (B) blank serum, (C) serum spiked with the three drugs at a level of 0.1 µg/mL. *Source*: Reproduced from *Talanta* [116] with permission from Elsevier.

CE‐C^4^D is also perfectly suited for the determination of small organic ions. This may be, for example, lactate as a marker of physical activity [114] or 3‐hydroxybutyrate (3‐HB) as an indicator of fasting [115]. Lactate was determined in less than a drop of capillary blood from a finger prick (diluted 1:20 with appropriate buffer solution) using a specifically designed device for separating blood plasma from blood cells. The plasma collection took approximately 20 s only, and it was subjected to a direct CE‐C^4^D analysis with sub‐minute separation times [114]. Lactate is usually present at mM concentration levels in blood, and CE‐C^4^D offers its sensitive determination in even 20‐fold diluted plasma samples. On the other hand, 3‐HB concentrations are one to two orders of magnitude lower, and improved detection sensitivity is essential for its determination, which can be achieved by large‐volume sample stacking (LVSS) using a covalently coated separation capillary. The developed method was used for 3‐HB monitoring during human fasting and demonstrated approximately 20‐fold higher 3‐HB concentrations after 60 h of fasting with a quick return to the normal physiological levels upon application of a high‐saccharide diet [115]. LVSS in combination with matrix precipitation by acetonitrile and the use of covalently coated separation capillaries may significantly improve the detection sensitivity of CE‐C^4^D. The three strategies were applied to the determination of other small ionic compounds in plasma and serum samples. These were, for example, zwitterionic antiepileptic drugs [116], broad‐spectrum antibiotics [117], symmetric and asymmetric dimethylarginine [113], and creatine, 2‐aminobutyric acid, acetyl‐carnitine and amino acids [118]. The determination of the antiepileptic drugs in serum samples using the three aforementioned strategies is depicted in Figure 7.

The blood precipitation with acetonitrile is, however, associated with sample dilution and reduction of analyte concentration in the resulting supernatant, which might have a direct consequence on the method sensitivity in specific cases. Tůma has, therefore, suggested an alternative technique for the determination of low‐abundant analytes in minute volumes of body fluid samples by using microdialysis (MD) [112]. Ten microlitres of body fluid was collected into a sampling capillary with an id of 430 µm. An MD hollow fibre with an od of 280 µm and a length of 5 cm was inserted into the sampling capillary and then filled with 2 µL of perfusate. Due to the minimum distance (sample to membrane to perfusate), the MD equilibrium was achieved in about 1 min. Concentrations of the analytes were identical on both sides of the membrane, and as the perfusate volume was only 2 µL, they were just slightly lower than in the original body fluid. The resulting microdialysate was then transferred into a CE micro‐insert for direct injection and analysis. The above‐reported concept was used for the determination of 20 amino acids in blood, plasma, tear and dried blood spot (DBS) samples [112]. A slightly modified MD procedure was also used for the determination of antibiotics in tissue samples of diabetic patients [117].

Collection and analysis of venous blood are accepted and widely used in clinical practice; nevertheless, their use also constitutes several drawbacks. The collection of venous blood requires a visit to a specialized medical centre, a trained phlebotomist, a dedicated shipment to the laboratory for the analysis, and often also deep‐freezing and storage. To reduce the patients’ discomfort and the shipment/storage costs and to eliminate the need for trained personnel, alternative biological samples have been increasingly used in clinical analysis in recent years. Collection of capillary blood from a finger/heel prick is significantly less invasive than phlebotomy, necessitates only µL volumes of blood, is suitable for self‐sampling and does not require cold‐chain shipment and storage after drying. Even more facile is the collection of saliva, sweat, urine and exhaled breath condensate (EBC), which are collected fully non‐invasively. Importantly, all these alternative biological samples are collected in µL volumes only and are perfectly compatible with the minimal requirements on sample volumes used for injections by CE instruments [119].

**FIGURE 8:**
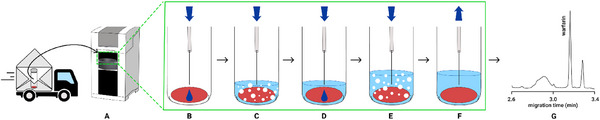
The concept of automated processing (A–E) and analysis (F–G) of a DBS by the commercial CE instrument. *Source*: Reproduced from *Angewandte Chemie International Edition* [120] with permission from Wiley.

The DBS technology has experienced tremendous development during the recent COVID‐19 pandemic as it enables a rather simple means of blood collection virtually at any time and any place. The DBS processing is, however, not as simple as its collection, and Kubáň and co‐workers have shown that a commercial CE instrument can be used as an all‐in‐one tool for the automated pretreatment and analysis of DBSs [120]. In their concept, the separation capillary of the CE instrument served for fluidic transfers of elution solutions to a DBS located in a CE vial, processing of the DBS, and also for the CE conditioning/flushing, direct injection of the resulting DBS eluate and separation. All these procedures were facilitated by the autosampler and the internal pneumatic and high‐voltage systems of the CE instrument and offered a fully automated pretreatment and analysis of up to 39 DBS samples in one sequence. The total analysis time was approximately 5 min per DBS (including DBS rehydration, matrix precipitation, analyte(s) elution, injection, separation and quantification) resulting in sample throughputs of up to 360 DBS samples per day. Fundamental principles of the automated DBS analysis by CE are shown in Figure 8. The novel concept was applied to the CE‐C^4^D determination of several small ionic compounds in DBS samples, including amino acids [121], creatinine [122] and uric acid [123]. Automation of the DBS processing and analysis was also achieved by the coupling of SI to CE. In the SI‐CE set‐up, the SI manifold served for the automated DBS processing and transfer of the DBS eluate to the commercial CE instrument for a fully automated analysis of inorganic cations and lithium, selected as the model clinical analyte [124].

Novel DBS technologies, that is, volumetric devices for DBS collection, have been adopted during the COVID‐19 pandemic and have addressed two major problems of DBS self‐sampling: (i) the collection of exact blood volumes independently of haematocrit and (ii) the non‐homogeneity effects. The new volumetric devices have significantly improved the potential of DBS samples for quantitative analyses. Nevertheless, their new designs and collection principles do not enable their automated processing by commercial DBS systems, and they are primarily analysed by a set of several manual processes. Dvořák and Kubáň have shown that the concept of the fully automated processing and analysis of DBSs by CE‐C^4^D is also suitable for the novel volumetric devices and have used it for the determination of creatinine as a clinically important marker of kidney malfunctioning [122]. Collection, transport and storage of dried biological material are not limited to blood, and any biological material can be used in the form of a dried spot. Quantitative aspects of self‐sampling dried urine spots (DUS) have been comprehensively examined and revealed a significant chromatographic effect of the standard cellulose‐based collection cards [125]. Several analytes (inorganic cations and creatinine detected by CE‐C^4^D) exhibited up to 20‐fold different concentrations in the central versus the peripheral part of the DUS (up to 3.75‐fold different concentrations to the parent liquid urine) suggesting that quantitative DUS analyses are possible only after sampling known volumes of urine and punching and eluting the whole DUSs.

Non‐invasive collection of body fluids is an obvious benefit for clinical subjects because it is not painful, is less stressful and can be done at home. The disadvantage of non‐invasively collected biological samples is that the clinical relevance of the obtained results has to be confirmed. This applies to urine, sweat, saliva and EBC as concentrations of analytes therein can vary during the day and also within days, and reference clinical ranges are not available or are by far less exact than for blood. Despite this, non‐invasive collection of body fluids is interesting for personalized healthcare and has attracted significant attention in clinical analysis in recent years. A temperature‐controlled sampler using open‐source technology has been designed and used for the collection of EBC samples with subsequent CE‐C^4^D determination of inorganic cations, inorganic anions and organic anions [126]. Inorganic cations and anions were determined in saliva samples using a separation capillary coated with three successive layers of various coating agents to avoid adsorption of proteinaceous matrix to the capillary walls [127]. Although electrolytes are present in high concentrations in all body fluids, low‐molecular‐mass thiols are present in µM concentrations and their determination without proper sample treatment might be challenging. Cysteine, homocysteine, glutathione and methionine were, therefore, determined in saliva samples by CE‐C^4^D after a selective extraction by gold nanoparticles and FASS [128]. A lab‐made portable CE‐C^4^D instrument was used for the determination of organic acids in infant stool samples [68].

Sweat, most easily collected by a skin wipe test, is also used in clinical analysis, particularly for the diagnosis of cystic fibrosis (CF). The standard procedures rely on the monitoring of chloride anion, which is considered the major biomarker of CF in sweat. CE‐C^4^D can be used as an alternative method for the determination of chloride in sweat samples; moreover, CE enables the simultaneous determination of other anions and cations in the eluate of the skin wipe offering additional information on the sweat ionic composition. Ďurč et al. have shown that the chloride:potassium and (chloride + sodium):potassium ratios determined by CE‐C^4^D were more specific for the diagnosis of CF in comparison to chloride as a single marker [129]. An alternative method for the sweat collection was also suggested using a 3D‐printed skin wash sampling. A milli‐fluidic device with a flow‐through channel was attached to the forearm and directly contacted the skin. DI water was flushed through the milli‐channel and was gradually enriched with sweat components on its contact with the forearm. The sample was collected on the opposite side of the device and used for the direct injection into a CE‐C^4^D instrument for the determination of inorganic anions and cations in the diagnosis of CF [130]. Sweat samples were also used for the demonstration of instrumental developments, including the fabrication of a low‐cost Arduino‐based data acquisition device [56] and an automated autosampler for lab‐made CE‐C^4^D systems [66].

Apart from the determination of inborn metabolic disorders, sweat composition can also be used for the monitoring of actual body status. Lactate is produced in the human body during physical activities, and its concentrations increase significantly compared to the standard levels during anaerobic sessions. Lactate is usually determined in blood samples for athletes’ performance monitoring, which requires blood sampling and might not be possible without dedicated equipment. Sweat has been reported to similarly reflect intensive exercises, and it also exhibits a significant increase in lactate concentrations during anaerobic physical activities. Lactate and other inorganic and organic anions can be easily determined by CE‐C^4^D, and two methods for lactate determination in sweat samples have been reported recently [131, 132] demonstrating an almost one order of magnitude increase in sweat lactate concentrations in exercise versus non‐exercise conditions [131].

Metabolic disorders, such as organic acidurias, are life‐threatening without an early diagnosis and adequate treatment. Their monitoring is often done by the analysis of urine samples, which usually contain higher concentrations of specific organic acids as potential markers for particular acidurias. A rapid and sensitive CE‐C^4^D method for the determination of orotic acid in urine samples was presented by Öztekin and co‐workers demonstrating sensitivity at a 0.014 mg/L concentration level after online sample preconcentration by LVSS [133]. Most acidurias are related to a single component that is accumulated in the body in one chemical form only. Nevertheless, some markers are chiral, and their chiral forms can play significant roles in the disorders. 2‐Hydroxyglutaric acid can be present in the d‐ and l‐form in humans, and two types of 2‐hydroxyglutaric aciduria are diagnosed. In order to determine the proper aciduria type, enantiomers of the marker have to be identified and quantified. CE is perfectly suited for chiral separations due to the minimal consumption of reagents and samples, and a CE‐C^4^D method for the determination of d‐ and l‐2‐hydroxyglutaric acid in urine was presented with the use of vancomycin as the chiral selector [134].

Urine has a longer detection time window for many exogenous compounds than blood and is, therefore, an important sample for doping control and clinical/toxicological analysis of drugs. However, urine contains high concentrations of endogenous matrix compounds, which might affect the determination of target analytes, and sample pretreatment is usually required before the analysis. SPE has been used for the pretreatment of urine and beverage samples before the CE‐C^4^D determination of γ‐hydroxybutyric acid (GHB), a popular club scene drug [135]. Quantitative analysis of GHB was possible down to 0.37 µM level, which was significantly below the endogenous GHB levels in urine. EME was online coupled to a lab‐made CE instrument for the pretreatment of urine and blood serum for the determination of methadone [136]. A flow‐through probe with a porous hollow fibre impregnated with a selective carrier (2‐nitrophenyl octyl ether) was used for the EME sample treatment. A syringe pump served for the online transfer of the clean and methadone‐enriched extract to the injection cross with the CE separation capillary and the grounding electrode. Flow‐gated injection and subsequent CE‐C^4^D separation of the extract demonstrated excellent sample clean‐up and sufficient methadone enrichment for its determination in real biological samples. Adamantane drugs are used for the treatment of serious diseases, such as hepatitis C, Parkinson's disease, Alzheimer's disease and influenza A. Their concentrations in body fluids are low (typically less than 1 µg/L), and their determination by CE techniques is not possible due to the limited CE sensitivity. Sample clean‐up and preconcentration employing ultrasound‐assisted dispersive liquid–liquid microextraction (UA‐DLLME) and FASS were, therefore, used for their CE‐C^4^D analysis. The method offered enrichment factors higher than 1342 resulting in LODs at the nM range, which were well below the therapeutic ranges for the drugs and enabled their therapeutic monitoring in human urine and serum samples [137]. A combined UA‐DLLME‐FASS approach was also used for the chiral analysis of phenothiazine derivatives in urine and serum samples [138]. The sample treatment was improved by the application of Tween 80 as a surfactant for the UA‐DLLME, whereas FASS was employed for the preferential injection of the charged analytes into the separation capillary. Hydroxypropyl‐β‐cyclodextrin was used for the chiral separation of the d‐ and l‐forms of five phenothiazine drugs with sub‐nM LODs and ensured their sensitive CE‐C^4^D determination in both body fluids. Creatinine is present in high concentrations in urine and is typically used for the normalization of the concentrations of other analytes. Thus, in comparison to all previously discussed analytes, there is no need for special sample treatment for creatinine measurements. A quick centrifugation of urine and 1:100 dilution with DI water were sufficient for the capillary isotachophoresis determination of creatinine as reported by Piestansky et al. [139].

#### 3.1.3 Environmental Samples

The quality of analytical data is primarily dependent on the quality of sampling and sample transport to the lab. In environmental analysis, the sampling/transport procedures are of utmost importance as many analytes are not stable in the samples even at the most deliberate transport conditions. Moreover, some environmental applications do not allow the transport of samples to the lab in a reasonable time [140], or in extra‐terrestrial analysis, this is not even possible [141]. On‐site analysis is, therefore, an obvious requirement for unbiased environmental monitoring in these situations. Nevertheless, on‐site applications of standard benchtop analytical instruments are not possible, and thus, portable analytical systems are essential. CE is undoubtedly the most amenable portable analytical separation technique for liquid samples due to its minimum power requirements, simple instrumentation and good tolerance to various sample matrices. Indeed, CE (particularly in combination with C^4^D) has been shown as a viable solution for on‐site analyses previously [142, 143, 144, 145], and the trend of the development of mobile CE instrumentation for environmental applications has continued in the reviewed period, too.

Drevinskas et al. demonstrated the possibility of determining volatile amines and organic acids as well as aerosol‐bound non‐volatile inorganic cations and proteins in air using a purpose‐made CE‐C^4^D instrument on a drone [72]. The lab‐on‐a‐drone concept can be especially helpful in life‐threatening situations when the sampling/analysis site presents a health risk for the analysts. For example, the analysis of nerve agents (and their degradation products) on a contaminated site represents a high risk of poisoning during sample collection and analysis, which might be minimized by the application of the lab‐on‐a‐drone [72]. In addition, the researchers at the Jet Propulsion Laboratory studied the separation of inorganic cations and amino acids [74], as well as of inorganic anions and mono‐, di‐ and tri‐carboxylic organic anions in highly saline samples (Mono Lake and cell culture media) [146] in view of extra‐terrestrial analysis of these species.

**FIGURE 9:**
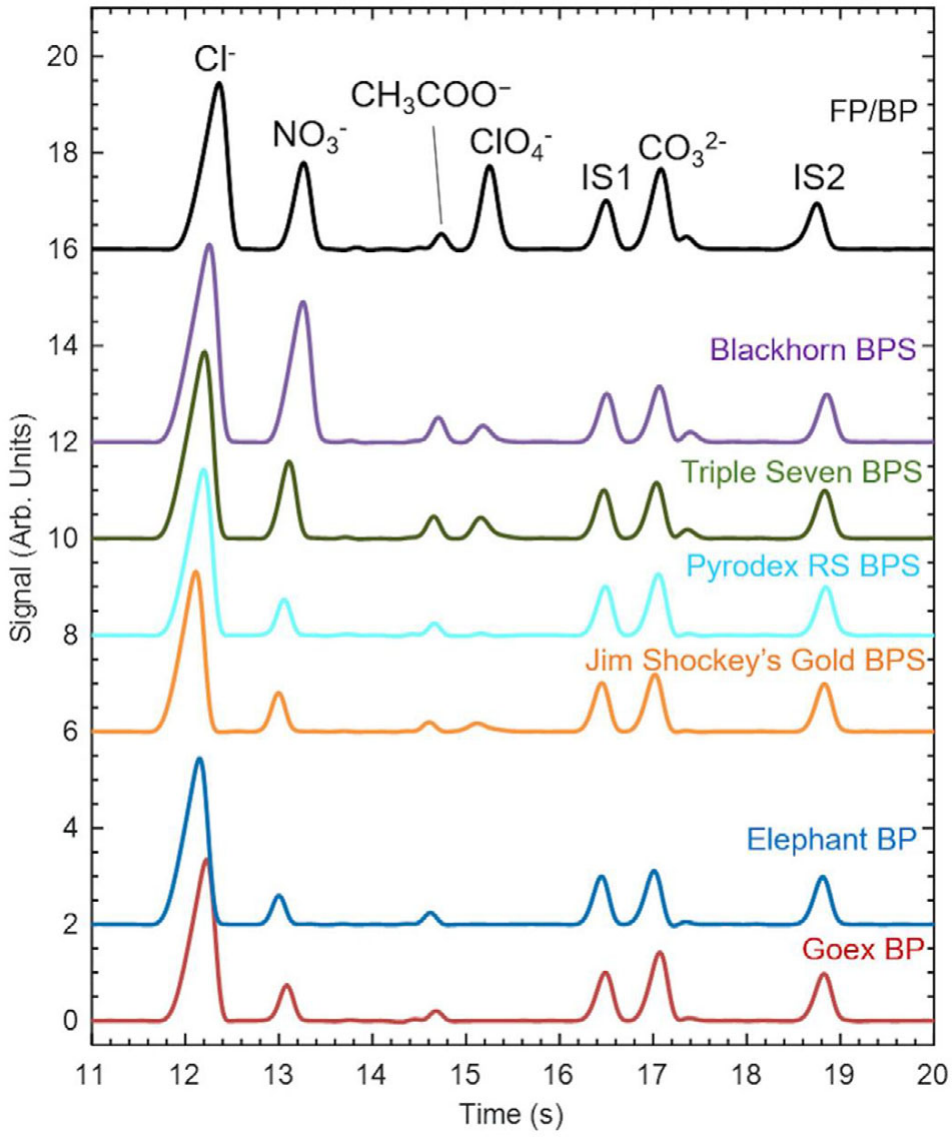
Representative electropherograms of inorganic and organic anions from surface wipes of flash powder (FP), black powder substitutes (BPS) and black powder (BP) samples; internal standards (IS1 and IS2) were not specified. *Source*: Reproduced from *Electrophoresis* [147] with permission from Wiley.

The commercial portable instrument from Grey Scan was launched for the detection of explosives [147]. It is based on the CE‐C^4^D determination of inorganic and organic anions collected with surface paper wipes (model ETD‐100) but is also available in a variant for the direct analysis of liquid samples (model ETD‐100L). The paper wipes are first eluted with 200 µL of elution solvent in 5 s, and the collected eluates are only filtered before their final injection into the separation capillary. All these procedures are done fully automatically using the all‐in‐one instrument and result in an analysis time of approximately 40 s per sample. The short and narrow capillary (30 cm, 25 µm id) enables the separation of major inorganic anions (chloride, carbonate and acetate) and anions relevant as potential markers of explosives (nitrate, chlorate and perchlorate) in less than 20 s; see Figure 9. Despite additional capillary flushing and equilibration (2–3 min) being necessary when a “positive” sample is detected, this technique shows an excellent sample throughput (as most samples are expected to be “negative”) and represents an attractive tool for on‐site screening of explosives [147]. Inorganic oxidizers (nitrate, chlorate and perchlorate) were also determined in various explosive materials by gradient elution moving boundary electrophoresis. No prior treatment except for dilution was required, and the technique was able to efficiently eliminate matrix interferences [148].

A lab‐made portable semiautomated instrument was used for the determination of alkyl sulphate surfactants in wastewater samples [149]. The analytes were first absorbed by Al_2_O_3_ from the samples and subsequently desorbed into a clean acceptor solution for their clean‐up/preconcentration and efficient/sensitive CE‐C^4^D determination. Other environmental applications included the determination of perfluoroalkyl substances in wastewater samples [150], total dissolved nitrogen content in surface water samples [151] and nutrients in soils [67, 152]. Novel on‐site sampling strategies for the subsequent CE‐C^4^D analyses of air and gaseous samples have also been suggested [153, 154].

#### 3.1.4 Industrial Samples and Standard Solutions

A portable CE‐C^4^D instrument was demonstrated for the on‐site determination of quaternary amines in industrial disinfectants [69]. C^4^D was also shown suitable for the visualization of charged species in NACE [155, 156] and in CE‐frontal analysis [157], which can be used for the determination of drug‐protein binding constants. CE‐C^4^D was used for the improvement of the qualitative and quantitative evaluation in CE. Do Lago and co‐workers demonstrated that better identification and quantification of analytes can be achieved when electropherograms are plotted as a function of time, charge and mobility [158] and that monitoring and integration of pressure during sample injection can improve the estimation of injected volumes and, thus, quantitative results [159].

Standard solutions were also used in the development of mathematical models for the correction of migration times, which demonstrated that by using 4 reference peaks, the migration time repeatability improved 15 times compared to that of non‐corrected migration times [160]. Different modes of air‐assisted flow‐gated injections in flow‐through interfaces were monitored by CE‐C^4^D. Flow‐gated injections are performed from extra‐small volumes of samples (less than 100 nL) transferred to the separation capillary through a transfer line formed from another low‐id capillary and result in injection repeatability better than 5% for axial as well as for perpendicular arrangements of the two capillaries [161].

A new method has been developed for the CE‐C^4^D determination of silver ions, which are used as a biocide for water treatment on National Aeronautics and Space Administration (NASA) vehicles and might require monitoring during its prolonged use in space [162]. C^4^D was also used for the CE determination of acidity constants and limiting electrophoretic mobilities of glyphosate and its transformation products [163], capillary isoelectric focussing of proteins in microfluidic set‐up using short capillaries [84], monitoring of migration of micellar segments in various MEKC systems [164] and as a novel detection tool for Taylor dispersion analysis of charged polymers [165].

### 3.2 Microchip Electrophoresis

**FIGURE 10:**
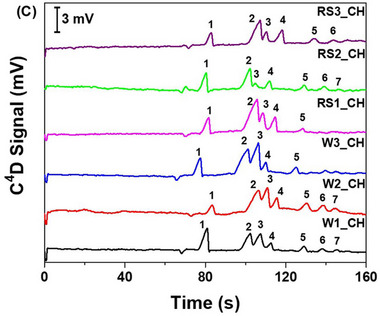
Electropherograms showing sulphate (1), tartrate (2), maleate (3), succinate (4), acetate (5), lactate (6) and phosphate (7) in white (W1–W3) and rose (RS1–RS3) wines. *Source*: Reproduced from *Talanta* [180] with permission from Elsevier.

A number of recent reports on applications of ME‐C^4^D have made use of the commercially available electrophoresis chips from Micronit and the matching measurement platform from eDAQ [174, 175, 179, 180, 181, 182, 183, 184, 185]. The use of commercial ME‐C^4^D devices enables precise control of the dimensions of electrodes and the detection gap, ease of operation and higher device‐to‐device reproducibility over the lab‐made systems. Consequently, several publications from recent years demonstrated separations and quantification of various analytes in real samples. These included the determination of amino acids and related compounds in food supplements [182, 185], inorganic and organic anions in wine [180] and whisky [183], naphthenic acids in water samples [181], alcoholic content in whiskeys [184] and drugs in food/clinical [179] and environmental [174, 175] samples. Figure 10 shows the ME‐C^4^D determination of inorganic and organic anions in white (W) and rose (RS) wine samples.

One of the general drawbacks of ME‐C^4^D in comparison to CE‐C^4^D is the lower sensitivity of the former, due to the different geometries of the detectors, and as a rule of thumb, one order of magnitude poorer sensitivity is typically achieved in ME‐C^4^D (compare the LOD data in Table 2 vs. Table 3). One possible strategy for sensitivity improvement is the use of FASS, which was applied to the determination of lead and cadmium ions in soil samples with sensitivity enhancement factors of 26 and 44 [186].

**TABLE 3:** Applications of C^4^D in ME.

Analytes	BGE composition	C^4^D parameters	Material	Mode	Sample type	LODs	refs.
L‐Carnosine	0.5 M acetic acid	eDAQ ER225, ET121; 40%, 400 kHz	PMMA	CZE	Health supplements	2.5 µM	[185]
Cl^−^, F^−^	15 mM His, 10 mM lactic acid, pH 5.8	eDAQ ER455; 60 *V*_pp_, 1100 kHz	Glass	CZE	Whisky	n.r.	[183]
Cl^−^, NO_3_^−^, SO_4_^2−^, PO_4_^3−^	n.r.	47 *V*_pp_, 200 kHz	n.r.	CZE	Soil samples	0.25–0.38 µM	[80]
Cl^−^, NO_3_^−^, SO_4_^2−^	MES/His, PVP/PTAE (concentrations n.r.)	50 *V*_pp_, 1000 kHz	PDMS	CZE	Soil samples	0.4 mg/L	[188]
Ethanol, butanol, pentanol	50 mM phosphate buffer, 30 mM SDS	eDAQ ER455; 20 *V*_pp_, 1200 kHz	Glass	MEKC	Whiskey	0.17%–0.5% (v/v)	[184]
Glutamic acid, trypsin inhibitor	MES/Tris, pH 8.1 Tris/HCl, pH 7.4 MES/His, pH 6.1 TEA/acetic acid, pH 10.6 (concentrations n.r.)	eDAQ ER225, ET121; 80 *V*_pp_, 600 kHz, 160 *V*_pp_, 200 kHz	Glass	CZE	Standard solutions	n.r.	[189]
l‐Histidine, β‐alanine	10 mM HIBA, 200 mM acetic acid, pH 2.62	eDAQ ER455; n.r., 800 kHz	Glass	CZE	Dietary supplements	4.2–5.2 µM	[182]
Inorganic and organic anions (12 anions)	30 mM MES, 15 mM His, 50 µM CTAB, pH 5.8	eDAQ ER455; 20 *V*_pp_, 1200 kHz	Glass	CZE	Wine	3.0–12.6 µM	[180]
K^+^, Ca^2+^, Mg^2+^, NH_4_^+^	20 mM MES, 20 mM His, 10 µM CTAB, 10 mM 18‐crown‐6	5 *V*_pp_, 20 *V*_pp_, 800 kHz	PMMA	CZE portable	Soil leachate	0.9–2.0 mg/L	[187]
K^+^, Na^+^, ASA	12.5 mM MES, 12.5 mM His	TraceDec	Silicon	CEC	Standard solutions	n.r.	[83]
K^+^, Na^+^, Li^+^	20 mM MES, 20 mM His, pH 6.0	5 *V*_pp_, 700 kHz	PMMA	CZE	Standard solutions	50–100 µM	[81]
K^+^, Na^+^, Li^+^	10 mM MES, 10 mM His, pH 6.15	60 *V*_pp_, 200 kHz	n.r.	CZE	Standard solutions	n.r.	[63]
K^+^, Na^+^, Li^+^	20 mM MES, 20 mM His, pH 6.0	3.5 *V*_pp_, 250 kHz	3D‐printed	CZE	Standard solutions	n.r.	[79]
K^+^, Na^+^, Li^+^	20 mM MES, 20 mM His, pH 6.1	1.8 *V*_pp_, 400 kHz	PMMA	CZE	Standard solutions	n.r.	[82]
K^+^, Na^+^, Li^+^	20 mM MES, 20 mM His	3.5 *V*_pp_, 268 kHz, 384 kHz	3D‐printed	CZE	Standard solutions	10.2–14.1 µM	[77]
K^+^, Na^+^, NH_4_^+^	n.r.	47 *V*_pp_, 200 kHz	n.r.	CZE	Soil samples	1.34–1.56 µM	[80]
K^+^, Na^+^, NH_4_^+^	MES/His, PVP/PTAE (concentrations n.r.)	50 *V*_pp_, 1000 kHz	PDMS	CZE	Soil samples	0.1–0.5 mg/L	[188]
Naphthenic acids (1‐naphthoic acid, benzoic acid, 9‐anthracene carboxylic acid)	10 mM carbonate buffer, pH 10.2	eDAQ ER455; 20 *V*_pp_, 1200 kHz	Glass	CZE	Produced water	4.7–7.7 µM	[181]
NO_3_^−^, H_2_PO_4_^−^	20 mM MES, 20 mM His, 10 µM CTAB, 10 mM 18‐crown‐6	5 *V*_pp_, 20 *V*_pp_, 800 kHz	PMMA	CZE portable	Soil leachate	3.1–4.85 mg/L	[187]
Nonsteroidal anti‐inflammatory drugs (Ibu, Ket, ASA, Dic)	20 mM His, 15 mM Tris, 2 mM HP‐β‐CD, 10% (v/v) methanol, pH 8.6	eDAQ ER455/225; 60 *V*_pp_, 700 kHz	Glass	SPE‐CZE	Environmental samples, waste water	0.156–0.625 mg/L	[174]
Paracetamol, *p*‐aminophenol	20 mM β‐alanine adjusted to pH 11 with NaOH, 14% (v/v) methanol	eDAQ ER455/225; 60 *V*_pp_, 1000 kHz	Glass	SPE‐CZE	Waste water	0.3125–0.625 mg/L	[175]
Pb^2+^, Cd^2+^	20 mM MES, 20 mM His, 10 µM CTAB, pH 6.1	n.r.	PMMA	FASS‐CZE	Soil leachate	n.r.	[186]
Scopolamine, butylscopolamine	40 mM butyric acid, 25 mM NaOH, pH 5.0	eDAQ ER455; 20 *V*_pp_, 1200 kHz	Glass	CZE	Beverages, urine, pharmaceuticals	1.1 µM	[179]

Abbreviations: ASA, acetylsalicylic acid; Dic, diclofenac; HIBA, α‐hydroxyisobutyric acid; His, l‐histidine; HP‐β‐CD, hydroxypropyl‐β‐cyclodextrin; Ibu, ibuprofen; Ket, ketoprofen; n.r., not reported; PTAE, phosphate/Trizma/acetate/ethylenediamine tetraacetic acid; TEA, triethylamine.

Electrophoretic separations and conductivity detection are techniques pertinent for portable operation, and many previous publications reported the development of portable CE‐ or ME‐C^4^D instruments [142, 143, 144, 145]. Clinical, industrial, or environmental assays may benefit from the on‐site analysis as many analytes and matrix components may decompose during sample transport. Nutrients in environmental samples are prone to such decomposition, and for example, concentrations of ammonium, nitrate and nitrite can change even in frozen samples. Compact, portable ME‐C^4^D instruments were, therefore, designed for the on‐site determination of ionic nutrients in soil samples [80, 187, 188]. In addition to the standard equipment needed for ME and C^4^D, the suitcase‐sized box also contained an electronic balance and a micro‐oscillator for weighing and homogenizing soil samples, respectively [80]. Powering of the instrument was possible by standard mains or by an outdoor power supply for on‐site operation. A complete list of ME‐C^4^D applications published in the reviewed period is given in Table 3, which contains comprehensive information on the analytical methods, including analyte name(s), BGE solution composition, C^4^D parameters, microchip material, CE separation mode, application area and LOD(s).

### 3.3 C^4^D for Other Analytical Applications

C^4^D has also been employed in methods other than CE and ME. Many processes are characterized by a change in the conductivity of corresponding reaction media, and even minor conductivity changes can be monitored with modern and highly sensitive C^4^Ds. Moreover, as C^4^D has no contact with the investigated media and is non‐destructive, it can even facilitate the detection of cells and bacteria.

C^4^D has, thus, been frequently used for the monitoring of two‐phase systems consisting of either liquid–liquid, gas–liquid or solid–liquid mixtures [190, 191, 192, 193, 194, 195, 196, 197, 198, 199, 200], conductivity measurements of stagnant or dynamic liquids [35, 88, 92, 201, 207] and the characterization of materials [208, 209]. Detection of charged species was also accomplished by the application of C^4^D in non‐separation (FIA) [86, 87, 210] and separation (HPLC, IC) [86, 211, 213] flow‐through techniques. The detection has been demonstrated for inorganic anions separated by IC [86], whereas the gradient programme [213] and the buffer concentration in a mobile phase [212] were monitored for HPLC separations. Conductivity measurements in contactless mode have also been employed for paper‐based analytical devices [214, 215], conductivity determination in solutions after electrophoretic titration [216, 217], liquid level measurements in infusion flasks [218], resistivity measurements during drilling in oil industry [219], water characteristics monitoring directly in pipelines [220], platelet aggregation monitoring [221] and monitoring bacterial activity in 8‐ and 32‐channel platforms [222, 223, 224].

## 4 Conclusions

CE‐C^4^D and ME‐C^4^D remain popular as evidenced by the large number of publications which have appeared in the last years. Most of these concern novel applications of CE‐C^4^D, most notably in the clinical field. The use of commercial detectors has become the norm demonstrating the consolidation of the C^4^D usage; these days, relatively few researchers are still building their own contactless conductivity detectors. Despite the inferior behaviour of planar microchip devices in terms of separation efficiency and LODs and the difficulties in their production, they still enjoy a degree of popularity, which might further grow due to the current availability of commercial ME‐C^4^D equipment. Although not covered in detail in this review, it is good to also see the continuous development of the uses of C^4^D in other analytical methods. The recent publications demonstrate the great power of C^4^D and confirm the excellent flexibility and variability of the detection scheme.

## Conflicts of Interest

The authors declare no conflicts of interest.

## Data Availability

The data that support the findings of this study are available from the corresponding author upon reasonable request.
